# Revisiting treatment-related cardiotoxicity in patients with malignant lymphoma—a review and prospects for the future

**DOI:** 10.3389/fcvm.2023.1243531

**Published:** 2023-08-30

**Authors:** Eva Rihackova, Michal Rihacek, Maria Vyskocilova, Dalibor Valik, Lubomir Elbl

**Affiliations:** ^1^ Department of Internal Medicine and Cardiology, University Hospital Brno and Faculty of Medicine of Masaryk University, Brno, Czech Republic; ^2^ Department of Laboratory Medicine, University Hospital Brno, Brno, Czech Republic; ^3^ Department of Laboratory Methods, Faculty of Medicine, Masaryk University, Brno, Czech Republic; ^4^ Department of Biochemistry, Faculty of Medicine, Masaryk University, Brno, Czech Republic; ^5^ Department of Pharmacology, Faculty of Medicine, Masaryk University, Brno, Czech Republic

**Keywords:** lymphoma, cardiotoxicity, chemotherapy, modern treatment, prevention, cardiac adverse events

## Abstract

Treatment of malignant lymphoma has for years been represented by many cardiotoxic agents especially anthracyclines, cyclophosphamide, and thoracic irradiation. Although they are in clinical practice for decades, the precise mechanism of cardiotoxicity and effective prevention is still part of the research. At this article we discuss most routinely used anti-cancer drugs in chemotherapeutic regiments for malignant lymphoma with the focus on novel insight on molecular mechanisms of cardiotoxicity. Understanding toxicity at molecular levels may unveil possible targets of cardioprotective supportive therapy or optimization of current therapeutic protocols. Additionally, we review novel specific targeted therapy and its challenges in cardio-oncology.

## 1. Introduction

Malignant lymphomas are neoplasms where the tumor cells are of lymphoid or histiocytic origin. 21st-century advances in understanding molecular pathology and immunephenotyping led to an important update in its WHO classification (1). Lymphomas are categorized into two main diagnostic subgroups according to their biological characteristics: Hodgkin's lymphoma (HL) and non-Hodgkin's lymphoma (NHL) (2). In NHL, one of the common treatment regimens used is R-CHOP consisting of rituximab, cyclophosphamide, doxorubicin, vincristine, prednisone) and there are various other regimens depending on molecular and antigenic properties of neoplastic elements (3). The most used regimens for HL are ABVD (doxorubicin, bleomycin, vinblastine, dacarbazine), BEACOPP (bleomycin, etoposide, doxorubicin, cyclophosphamide, vincristine, procarbazine, prednisone) and escalated BECOPP with higher doses of cyclophosphamide, doxorubicin, etoposide and with granulocyte colony-stimulating factor (4, 5). Anti-cancer treatment regularly combines chemotherapy with local irradiation (5). Wide range of other regimens are available, and selection depends on the type and severity of the disease, the clinical condition of a patient, or the presence of relapse.

Cardiovascular adverse events related to anti-cancer therapy generally include heart failure, myocarditis, vascular toxicities, hypertension, cardiac arrhythmias, corrected QT prolongation, and pericardial vascular heart disease. Association between therapeutic modality (anti-cancer drugs or radiotherapy) and adverse events is either established or still being investigated (see Table 1). The exact definitions of entities listed above are defined in ESC Guidelines on cardio-oncology 2022 (6). According to the meta-analysis of Boyne et al., both HL and NHL long-term survivors suffer from increased risk of death from cardiovascular disease (7.3 and 5.35 times higher, respectively) compared to the general population (7). Among HL survivors treated before the age of 25, the risk of a cardiovascular event was even higher and the 40-year cumulative incidence of cardiovascular disease was 50% in this population (7, 8). With regards to a high survival rate of HL patients, this topic has become an emerging issue in after-care, especially for HL patients treated at a younger age. Treatment-related cardiotoxicity is classified as acute and chronic, where chronic is divided into two main subcategories: early-onset (type I) and late-onset (type II). Type I occurs within one year after chemotherapy cessation. Type II cardiotoxicity is detected after the first year with an unlimited timeframe and sometimes may be observed even decades after discontinuation of chemotherapy (9).

**Table 1:** Association between therapeutic modality (anti-cancer chemotherapy or radiotherapy) and adverse events.

	Valvular damage	Arrhythmia	Takotsubo cardiomyopathy	Myocardial infarction	↓LVEF	Myocarditis	Pericardial disease	Vascular toxicity
DOX	(24)	(19)	(20, 21)	(24)	(22)	(18)	(24)	(18)
CTX		(92)		(99)	(90, 93, 95)	(90, 91)	(90)	
Rituximab		(140)	(137)	(141)	(144)			
Platinum-based drugs		(130, 131)		(136)	(135)			(125)
Vinca alkaloids				(148, 149)				
Bleomycin				(155, 156)				(153)
Dacarbazine								
Etoposide		(164, 165)		(162, 163)				
Procarbazine								
Prednisone		(171)						
Thoracic irradiation	(182)	(191)	(192)	(180)	(193)		(180)	(184)

The relation was determined either in clinical trials (pink squares) or in case reports (yellow squares) or not evaluated/discovered (white squares).

### 1.1. Anthracyclines

Anthracycline drug family discovery is dated in the 1950s with daunorubicin isolation from *Streptomyces peucetius* (10). Subsequently, a derivative of daunorubicin called Adriamycin, later renamed doxorubicin (DOX), was synthesized and both of them proved to be effective anti-tumor agents (11, 12). DOX is one of the most potent drugs used in the treatment of both NHL and HL (13).

Anthracycline anti-tumor effect depends on several mechanisms such as apoptosis induction via inhibition of topoisomerase II (TOP2), intercalation into the deoxyribonucleic acid (DNA) leading to an inhibition of macromolecules synthesis, or production of reactive oxygen species (ROS) causing DNA damage or lipid peroxidation (14).

Anti-tumor effect of anthracyclines is accompanied by dose-dependent cardiac toxicity that has been thoroughly investigated in studies trying to establish a safe dose of anthracyclines (15). Cardiotoxic properties are the main limit of its use in elderly lymphoma patients and in patients with history of cardiac disease (13).

Cardiotoxicity of DOX clinically presents as both acute and chronic. Acute cardiotoxicity may emerge as acute and usually reversible heart failure and/or acute arrhythmogenicity which usually manifests as premature ventricular beats observed after 10 min to 24 h following infusion of DOX (12, 16). Clinical manifestation of acute cardiotoxicity includes toxic myocarditis with cardiomyocyte impairment, inflammatory reaction and interstitial edema (17). Vascular toxicity may be caused by increased platelet adhesion with endothelial cells leading to formation of microthrombi and compromised blood flow (18). Anthracyclines reduce myocardial repolarization reserve which increases the risk of Torsade de Pointes (especially in combination with other QT-prolonging agents and hypokalemia) (19). Furthermore, takotsubo cardiomyopathy in a 24-year-old and 53-year-old man treated with anthracyclines was reported (20, 21).

Regarding chronic anthracyclines cardiotoxicity, a large study from the year 2003 revealed that 5%, 16%, and 26% of patients experienced DOX-related heart failure at cumulative doses of 400, 500, and 550 mg/m^2^ respectively. Also, the authors postulated age as a risk factor for DOX-related heart failure (22). Based on this study the upper limit for a cumulative dose of anthracyclines 400–450 mg/m^2^ was established. In a prospective study of 120 patients treated for advanced breast cancer, epirubicin induced a slowly progressing decrease of cardiac function continuing years after treatment cessation, 20% of patients progressed into chronic heart failure in 3 years after cumulative dose 850–1,000 mg/m^2^ of epirubicin (23).

Chronic cardiotoxicity represents a growing concern, especially in childhood cancer survivor cohort. Survivors were significantly more likely to develop congestive heart failure, myocardial infarction, pericardial disease, or even valvular pathology than their healthy siblings (cumulative dose of DOX 250 mg/m^2^ or higher increased relative hazard o cardiac adverse event by 2–5 times) (24). Another study consisting of 830 children treated with a cumulative dose of DOX 300 mg/m^2^ presented a 10% incidence of anthracycline-induced chronic heart failure (25). All these data support long-term close cardiac monitoring after discontinuation of anthracycline therapy. Risk factors for anthracycline-induced cardiotoxicity include anthracycline dose, female gender, concomitant irradiation, age, genetic factors, concomitance with trastuzumab administration, and anticancer treatment during childhood (22, 26–28).

#### 1.1.1. Mechanisms of anthracycline cardiac toxicity

Anthracycline cardiotoxicity has been a subject of research for more than 70 years, however, the exact mechanism has not yet been clarified. Early research focused on ROS-mediated cell and DNA damage. Antineoplastic agents exert their main toxic effects in tissues composed of rapidly dividing cells, however, myocardial cells have limited regenerative capability resulting in irreversible damage (29). Initial studies showed that the main cause of cardiotoxicity is oxidative stress caused by iron-anthracycline complexes that resulted in lipid peroxidation and cell membrane damage. The high sensitivity of myocardium to oxidative stress may be attributed to lower activities of ROS depleting protective mechanisms such as catalase, DOX-induced depletion of glutathione peroxidase, high myocardial metabolic activity, and high concentration of cardiolipin with positive affinity to anthracyclines (30–33).

Cardiotoxicity of anthracyclines was thought to be based on inhibition of the reduction of NAD^+^ to NADH+H^+^ during reverse electron transport in mitochondrial respiratory complex I. Furthermore, DOX caused a reduction of molecular oxygen (to O^2−^) followed by the rise of oxygen consumption by anthracycline semiquinone radicals (34). Oxidative stress leads to the activation of several apoptotic pathways such as the p53 pathway and p38 mitogen-activated protein kinase (MAPK) (35, 36). Cytochrome c is located in the inner membrane of mitochondrion and administration of DOX is causing cytochrome c release, which leads to activation of procaspase-9 and generating caspase-9 which proteolytically activates caspase-3 responsible for DNA fragmentation and apoptosis. According to many studies, DOX can trigger intrinsic, extrinsic, and endoplasmic reticulum-associated apoptotic pathways (37, 38).

**Figure 1:**
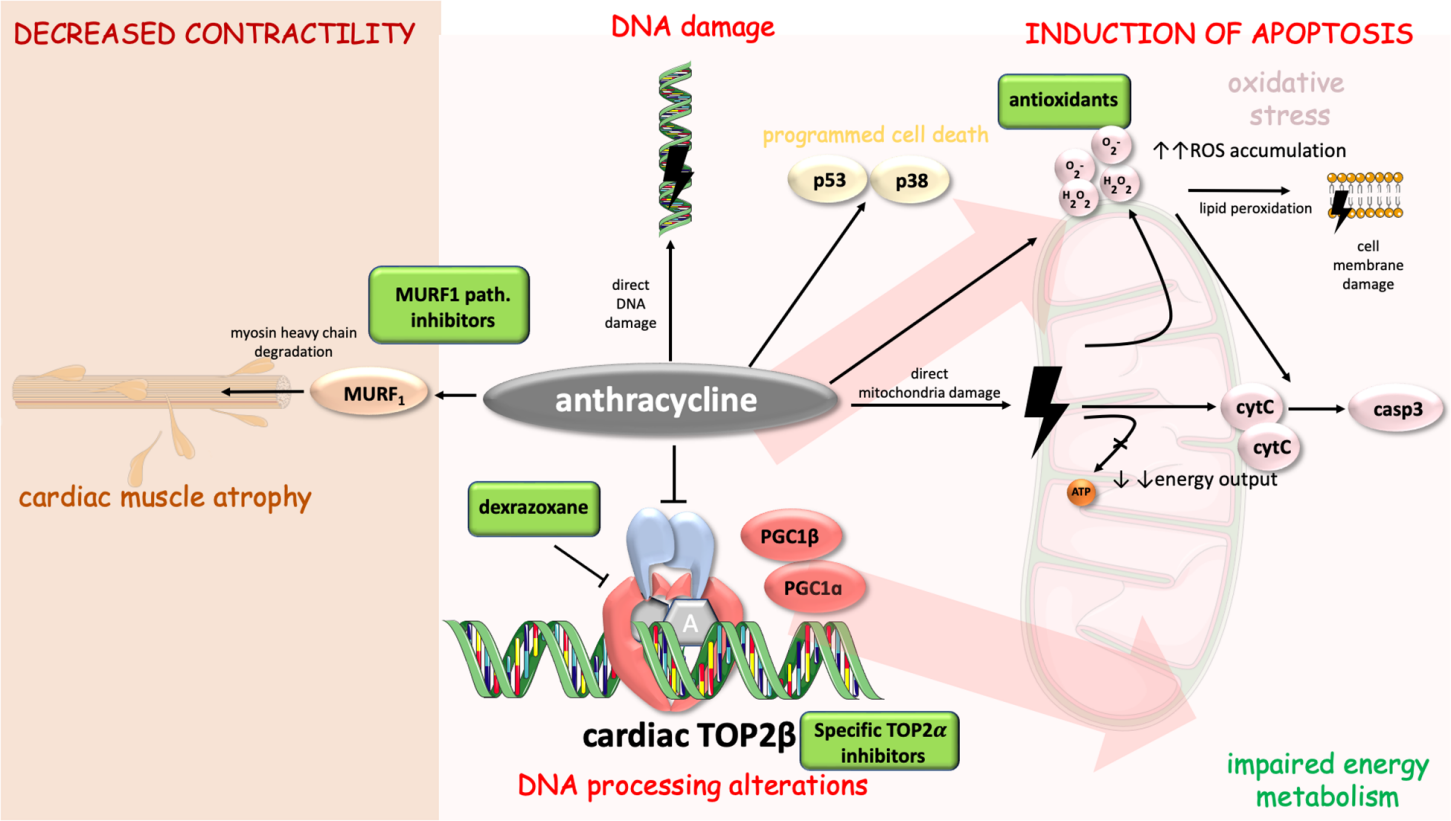
Mechanisms of anthracycline-induced cardiotoxicity with highlighted possible therapeutic targets. Anthracycline cytotoxic effects in cell include DNA alterations [direct damage and processing alterations through inhibition of cardiac TOP2beta (14)], energy metabolism impairment, induction of apoptosis [via casp3, p53 and p38 pathway (25, 35, 39)], and decreased contractility [involvement of MURF1 pathway and myosin heavy chain degradation (56, 57)]. Cell parts illustrations were implemented from free Servier repository available at: https://smart.servier.com/.

Furthermore, adaptive responses to DOX were discovered. Administration of DOX increases copper-zinc superoxide dismutase (supporting the theory about DOX-induced superoxide radical production). Additionally, an increase in BCL2:BAX ratio was observed (as an adaptation to antioxidant stress). All these findings support the theory that DOX induces oxidative stress and mitochondria-mediated apoptosis which goes hand in hand with adaptive responses to protect cardiac myocytes (39). Mechanisms of anthracycline-induced cardiotoxicity with possible therapeutic targets are displayed in Figure 1.

##### 1.1.1.1. Mitochondrial dysfunction

The proposed mechanism of chronic cardiotoxicity is a qualitative and quantitative injury of mitochondrial DNA (via impaired respiratory chain and increased production of ROS). These effects accumulate over time even in the absence of anthracycline exposure (40). DOX also stabilizes DNA-TOP2 cleavable complexes, resulting in double-strand breaks (41). This mechanism may lead to a decrease of mitochondrial DNA content and a rise in lactate concentration (42). Altering mitochondrial function may result in cardiomyocyte death and impairment of high-energy phosphate metabolism even in the absence of cardiomyopathy (43, 44).

##### 1.1.1.2. Role of topoisomerase II beta

Type II topoisomerases are divided into two subfamilies (α and β) (41). More recently, studies were focused on the possible role of Topoisomerase II β (TOP2β) in DOX-induced cardiotoxicity. While Topoisomerase II α (TOP2α) is present dominantly in proliferating cells, TOP2β is found mostly in quiescent cells including cardiomyocytes (45). Peroxisome proliferator-activated receptor gamma coactivator-1 α and β (PGC1α and PGC1β) play an important role in energy processes in mitochondria (46). Expression of PGC1α is decreased in failing human hearts (47). DOX-TOP2β complexes inhibit transcription of the genes PGC1α and PGC1β, which may result in impairment of energy and antioxidative metabolism according to several studies, and lead to mitochondrial damage (48, 49).

Mice with cardiomyocyte-specific deletion of the TOP2β do not develop DOX-induced heart failure, therefore, it is suggested that TOP2β plays a key role in DOX-mediated cardiotoxicity (48). Furthermore, according to mice models, ROS production and p53 activation seem to be TOP2β-dependent (50). To conclude, the presence of TOP2β is essential for DOX-mediated double-strand breaks, activation of apoptotic pathways, and impairment of mitochondrial function and ROS production(51).

##### 1.1.1.3. Role of muscle ring finger −1 and myocardial atrophy

Recently, several new studies focused on a myocardial mass evaluated by cardiovascular magnetic resonance (CMR). These studies discovered that left ventricular (LV) mass declines after anthracycline chemotherapy. However, this observation was not confirmed in patients receiving chemotherapy without anthracyclines (52). It seems that the decline in LV mass results from cardiomyocyte atrophy (reduction in a cardiomyocyte size) (53). MuRF1 (muscle ring finger −1) is a ubiquitin ligase marking defective proteins for degradation in proteasome and is essential for the development of cardiac atrophy (54, 55). DOX-treated mice developed dose-dependent upregulation of the ubiquitin ligase MuRF1 that was responsible for cardiac atrophy (55). MuRF1 pathway may therefore be regarded a candidate target to prevent DOX-mediated cardiac atrophy.

##### 1.1.1.4. Specifics of DOX-mediated cardiotoxicity in childhood

Pathophysiological mechanisms underlying cardiac damage during anthracycline therapy in childhood result from organ system development and growth. One of these proposed mechanisms of chronic cardiotoxicity in childhood cancer survivors is through DOX-mediated reduction of proliferation and differentiation of the progenitor cells and impairment of vascular development with decreased capillary density. These conditions may result in the heart being more susceptible to stress during adulthood (58).

##### 1.1.1.5. Other possible mechanisms of anthracycline cardiotoxicity

Other mechanisms of cardiotoxicity include direct DNA damage, disruption of the sarcomere protein titin (involved in force regulation of sarcomeres), and alterations in phospholipid content (32, 59). Electron microscopic examination shows a myofibrillar loss, vacuolar degeneration, and nuclei exhibit chromatin disorganization leading to cell death (60).

#### 1.1.2. Prevention of cardiotoxicity

##### 1.1.2.1. Antioxidants, apoptotic pathway inhibitors, angiotensin converting enzyme inhibitors (ACE-i), beta blockers

Initial preventive measures were focused on decreasing oxidative stress in cardiomyocytes. Various animal and cell culture studies have tried to exploit relevant pathways of anthracycline-mediated cardiotoxicity. Carvedilol is an adrenergic-blocking agent with antioxidant activity. Possible cardioprotective properties of carvedilol were studied in cultured cardiac muscle cells and pre-treatment with carvedilol significantly attenuated the production of ROS and DNA fragmentation but atenolol (with no antioxidative effect) did not possess cardioprotective properties (61). DOX induces cyclooxygenase-2 activity, which is associated with a cardiac injury that could be prevented by an administration of prostacyclin according to an *in vivo* study by Down et al. (62) on murine models.

Apoptosis of bovine aortic cells exposed to DOX was accompanied by a significant increase in cellular iron uptake and activation of iron regulatory protein 1, the latter mediated by the transferrin receptor. Iron uptake, cell apoptosis, and intracellular oxidant formation were significantly reduced in the presence of an anti-transferrin receptor antibody. Similar effects were observed with iron chelators (63). Another possible target is Protein kinase B (also known as Akt), which is a serine/threonine kinase promoting anti-apoptotic signals (64). In animal models, intracoronary adenoviral vector Akt 1 gene delivery resulted in the inhibition of DOX-induced reduction in cardiac function (65, 66).

The administration of probucol, a molecule with an antioxidant effect, prevented the decline of cardiac function caused by DOX. Probucol prevented changes (phosphorylation) of pro-apoptotically acting MAPK (67).

Unfortunately, from many possible cardioprotective agents (N-acetylcysteine, phenethylamine, coenzyme Q10, vitamin E, C, L-carnitine, carvedilol, amifostine, and dexrazoxane) only dexrazoxane statistically proved cardioprotective effect in humans (68).

In a prospective, randomized, controlled study possible cardioprotective long-term effects in lymphoma patients treated with ACE-i (enalapril) or beta-blocker (metoprolol) or placebo were examined, but they did not prove statistically significant effects (69).

Another randomized, controlled, double blind clinical trial was PRADA study on candesartan and metoprolol effect on cardiac dysfunction during adjuvant breast cancer therapy. Patients in the control group had 2.6% decrease in LVEF. On the contrary, patients receiving candesartan had 0.8% decline in LVEF. Surprisingly, there was no effect on global longitudinal strain, diastolic LV function, brain natriuretic peptide or troponin levels. Metoprolol did not provide positive effect on LVEF (70). Currently, another clinical trial investigating possible cardioprotective properties of betablockers and ACE inhibitors in breast cancer patients treated with anthracyclines, is running (SAFE study) (71). However, clinical studies fail to provide strong evidence on benefits of ACEi and BB in prevention of anthracycline cardiotoxicity.

##### 1.1.2.2. Natural bioactive compounds (NBACs)

Natural bioactive compounds (NBACs) have recently been of high interest for their possible medical effects. On contrast of synthetic drugs, this class of molecules is of natural origin (e.g., terpenes, flavonoids, alkaloids etc.). Elfadadny et al. (72) reviewed possible relationship between DOX and various NBACs regarding anti-tumour regulatory effects and toxicity protection. Through the reduction of ROS synthesis and increased activity of antioxidant enzymes, NBACs could mitigate DOX-induced cardiotoxicity. Moreover, via NBACs high affinity to TOP2α, NBACs may augment the anti-tumor effect. However clinical application of these compounds in therapeutic protocols should be supported by randomized clinical trials in the future (72).

##### 1.1.2.3. Dexrazoxane

Considering that cardiotoxicity is mediated by the Fenton reaction, many studies were focused on studying chelating agents with an assumption of their cardioprotective properties. In the study by Swain et al. (73), dexrazoxane (chelating agent) was administered after a cumulative doxorubicin dose of 300 mg/m^2^ in patients treated for advanced breast cancer (in combination with 5-fluorouracil and cyclophosphamide). The overall incidence of heart failure was 3% in the group receiving dexrazoxane vs. 22% in the placebo group. Moreover, dexrazoxane was confirmed to reduce troponin elevation and late cardiotoxicity in children treated for acute lymphoblastic leukemia (74).

Interestingly, another chelating agent deferasirox did not possess cardioprotective properties, suggesting that iron chelation might not be the main pathophysiological mechanism responsible for dexrazoxane's protective effect (75). It has been proposed that the main effect may be exerted through blockage of DOX interference with TOP2β (76). According to Lyu et al. (76), dexrazoxane changes the configuration of TOP2β thus preventing binding of DOX. Consequently, these findings may result in an apprehension of dexrazoxane mitigating anticancer effects, but in the meta-analysis, dexrazoxane did not alter the time to disease progression or survival rate. Current recommendations include dexrazoxane following DOX dose of 300 mg/m^2^ or higher (77, 78).

##### 1.1.2.4. Liposomal doxorubicin

Using liposomal encapsulation of DOX affects its pharmacokinetics and tissue distribution. Liposomal DOX cannot pass through the vessel wall with tight capillary junctions in healthy organs such as the heart but passes easily through the leaky endothelium in tumor tissue. Additionally, tumor tissue lacks functional lymphatic drainage, which results in liposomal DOX accumulation (79). Liposomal doxorubicin may be used in pegylated (more frequent) and non-pegylated forms. These forms increase the half-life of the original drug (80). These forms proved to be comparably efficient with significantly reduced cardiotoxicity, but their applicability is limited by the cost (81).

#### 1.1.3. Secondary prevention

Cardiac damage may be diagnosed or monitored by laboratory parameters or imaging methods (CMR, echocardiography). Laboratory parameters for early detection of left ventricular dysfunction include troponin and N-terminal pro B-type natriuretic peptide (NT-proBNP) (82–84).

Many parameters obtained from imaging methods were evaluated with promising results including a new parameter, the myocardial work which integrates cardiac deformation and LV pressure. In a study of 130 patients with NHL scheduled for R-CHOP, myocardial work proved to be appropriate for diagnosing subclinical cardiotoxicity and predicting left ventricle ejection fraction (LVEF) decline (85). Current standards for the identification of DOX-induced cardiotoxicity include an evaluation with LVEF and global longitudinal strain. However, other parameters are discussed including LV mass [in the study from Jordan et al. (52) evaluated from CMR] which proved to be a better predictor of heart failure symptomatology than LVEF (52, 86). The study of Jordan et al. (52) also proposed that myocellular dysfunction may not be pathophysiologically linked to impaired adaptive remodeling during anthracycline chemotherapy.

##### 1.1.3.1. Prompt initiation of heart failure therapy

According to study of 201 patients with anthracycline-induced cardiomyopathy, early detection of decreased ejection fraction (LVEF below 45%) with prompt initiation of heart failure therapy with ACE-i (enalapril) and carvedilol led to LVEF recovery and cardiac event reduction. On the contrary, the initiation of treatment of CHF in the time greater than 6 months after discontinuation of chemotherapy resulted in permanently reduced LVEF in all patients (87).

### 1.2. Cyclophosphamide

Cyclophosphamide (CTX) acts as an alkylating agent with antineoplastic, immunosuppressive, and immunomodulatory properties. CTX is a part of many therapeutic protocols used during stem cell transplantation, anti-cancer therapy, and several refractory autoimmune conditions treatment (88). CTX is used in the treatment of advanced stages of malignant lymphoid neoplasms. These may include HL and NHL (e.g., lymphocytic lymphoma, small lymphocytic lymphoma, Burkitt lymphoma), and multiple myeloma (89).

**Figure 2:**
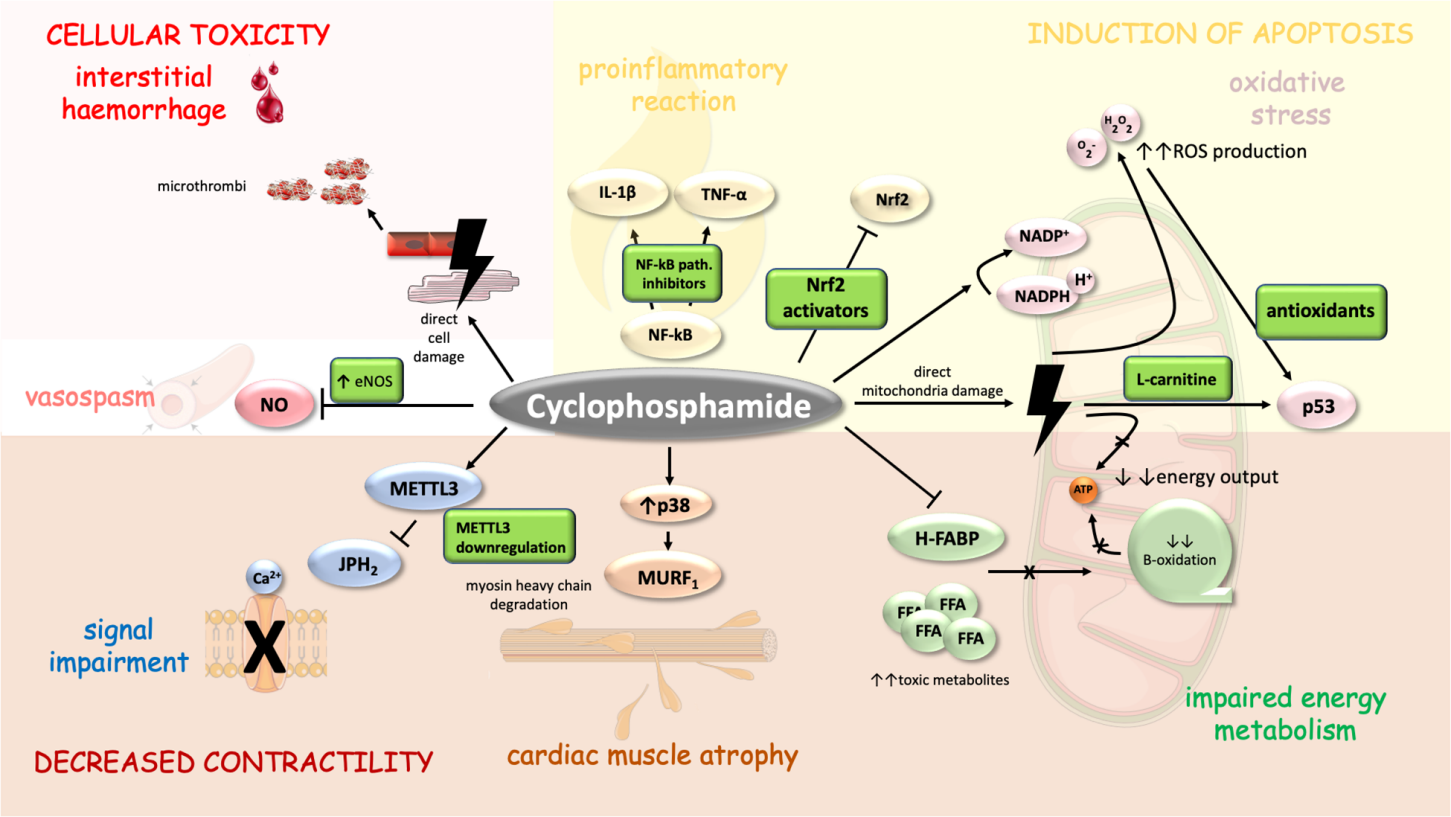
Mechanism of CTX-induced cardiomyopathy and its possible pharmacoprevention. CTX cytotoxic properties are similar to those of anthracyclines to a certain degree as both drugs direct several similar cellular compartments including mitochondria (energy metabolism) (99) and myosin heavy chains (MURF1) (111). Moreover, CTX cardiac adverse events are mediated through signal impairment (METTL3 downregulation), vasospasm (inhibition of NOS), interstitial haemorrhage (induced through direct cell damage and formation of microthrombi), inflammation (upregulation of NF-kappa-B), oxidative stress and apoptosis (103–105). Cell parts illustrations were implemented from free Servier repository available at: https://smart.servier.com/.

High-dose CTX may cause pericardial effusions, pericardial tamponade, CHF, and acute hemorrhagic myopericarditis with myocardial thickening (caused by intramyocardial extravasation of blood, fibrin, or fibrin-platelet microthrombi in capillaries, and fibrin strands in interstitium—see Figure 2) and progressive ventricular dysfunction (90, 91). In addition, in a case study, CTX administration led to atrial fibrillation with rapid ventricular rate was described (92). Systolic dysfunction may develop from a single dose of high-dose CTX and is less dependent on a cumulative dose. Systolic dysfunction usually occurs 5–16 days after initiation of CTX therapy and is potentially reversible (90, 93–97),. Depression of ECG voltage, ST abnormalities, systolic dysfunction, or increase in left ventricle diastolic/systolic diameter on echocardiography and troponin elevation may predict cardiac injury (90, 95).

Cardiotoxic properties are mainly associated with high doses of CTX 120–200 mg/kg usually administered over 2–5 days (90, 98, 99). Dose calculation based on body surface area proved to be a better cardiotoxicity predictor than a calculation based on weight. The predicted safe dose of CTX is 1,55 g/m^2^/dose (93).

High-dose CTX regimens around 200 mg/kg are included in bone marrow transplantation, as escalation to the upper limit significantly reduces the risk of relapse (100). However, the incidence of heart failure following bone marrow transplantation with CTX regimen reaches 20% and mortality about 8% (95).

In refractory autoimmune conditions such as refractory systemic lupus erythematosus (with or without subsequent stem cell transplant), rheumatoid arthritis, multiple sclerosis or hemolytic anemia etc., high-dose CTX (200 mg/kg) is a part of several treatment protocols (101–106). Risk factors for CTX-mediated cardiotoxicity include lymphoma patients, concomitant use of anthracyclines, mediastinal irradiation, and patients in higher risk of developing ischemic heart disease (88).

#### 1.2.1. Mechanism of cardiotoxicity

CTX is a prodrug metabolized by hepatic cytochrome C 450 into 4-hydroxycyclophosphamide and aldocyclophosphamide, which decomposes into cytotoxic phosphoramide mustard and acrolein (107). While phosphoramide is responsible for therapeutic effect (acts on seven-guanine residues of DNA leading to intrastrand and interstrand cross-links and cell death), acrolein may act as toxic mainly in the cardiovascular system interfering with the antioxidative metabolism resulting in elevated ROS production and drop of endothelial nitric oxide (NO) formation (99, 108–110). Additionally, toxic acrolein potential includes hemorrhagic cystitis and other toxicities linked to CTX such as gonadal toxicity, carcinogenesis, and bone marrow suppression (111). Some authors suggest that CTX metabolites may even cause direct damage to the endothelial cells and myocardium resulting in edema, interstitial hemorrhage and the formation of microthrombi (88). Endothelial cells are susceptible to CTX toxic effects, possibly due to their high proliferation rate and CTX -induced drop of production of endothelial NO leading to endothelial dysfunction (88).

#### 1.2.2. Molecular pathways impaired

There are studies demonstrating activation of the p38-MAPK pathway, through which acrolein may act as an upregulator of E3 ligases and MuRF1. This results in the degradation of myosin heavy chain (56, 57). Acrolein preferentially binds to nucleolar ribosomal DNA and induces oxidative stress-mediated damage and provokes ribosomal stress responses by activation of p53 (112). CTX also triggers various proinflammatory and proapoptotic mediator responses such as those mediated by nuclear factor-kappaB (NF-κB), cyclooxygenase-2, tumor necrosis factor-α (TNF-α) and interleukin-1β (IL-1β) (113). These pathways are potential targets to antagonize CTX -induced cardiotoxicity (114, 115).

CTX therapy is associated with reduced expression of heart-type fatty acid-binding proteins (H-FABP) with a consecutive decrease of mitochondrial transport and the oxidation of long-chain fatty acid (LCFA) (116). Oxidative metabolism of free fatty acids (FFA) provides about 70% of the energy required for the normal function of the myocardium and its decline leads to energy deficiency with subsequent accumulation of FFA toxic metabolites (117). Nevertheless, daily supplementation of L-carnitine might possibly reverse these effects (117).

By these mechanisms CTX and its main metabolite acrolein cause vasospasm, myocyte dysfunction, and necrosis/apoptosis which may lead to heart failure (109). Recently Zhu et col. demonstrated that CTX induces RNA N6-methyladenosine (m6A) modification by upregulating methyltransferases METTL3 expression and suppressing junktophillin-2 (JPH2) expression. By this mechanism CTX causes calcium signaling dysregulation and cardiac dysfunction and this pathway may possibly represent target for cardioprotective therapy (118).

Nuclear factor erythroid 2-related factor 2 pathway (Nrf2) impairs both oxidative stress and inflammation response) and according to some authors it is one of the main pathways by which CTX could cause cardiotoxicity (119).

#### 1.2.3. Prevention

Several molecules exhibited antioxidative properties that mitigated CTX -induced cardiotoxicity in *in vivo* animal models. Kolaviron (mixture of flavonoids with antioxidant and membrane stabilizing effect) administered 14 days prior to CTX treatment reduced CTX -mediated alteration of cardiac structure and metabolism (108). Furthermore, the combination of curcumine (cardioprotective phytoconstituent) and piperine (bio-enhancer) exhibited significant protection against CYP-induced myocardial toxicity and pre-treatment with N-acetylcysteine provides some cardioprotective effect with no alteration of CTX metabolism and its therapeutic efficacy (120, 121).

According to the preclinical studies above, the use of antioxidants to reduce ROS generation may suppress cardiotoxic adverse events. However, further human *in vivo* studies are needed to show their potential therapeutic efficacy (111). Another potential cardioprotective agent seems to be nicoradil which possess potassium channel opening effect, stimulates eNOS gene expression and has anti-inflammatory and antiapoptotic properties (122). Stern et col published a study on a multicellular coculture [hepatocytes, cardiomyocytes, triple negative breast cancer (TNBC) cells] with CN06 dual activator of Nrf2 and constitutive androstane receptor for TNBC. Via selectively activation of NrF2 antioxidant signaling in cardiomyocytes but not in TNBC cells they repressed DOX induced cardiotoxicity (with reduced apoptosis and enhanced kinetics of contraction) (123).

### 1.3. Platinum-based chemotherapy agents

These agents possess anti-tumor properties through crosslinking of DNA and formation of DNA adducts that activate apoptotic pathways including p53, p73, and MAPK (124). One of the main adverse effects is vascular toxicity linked to obesity and hypertension (125). Cisplatin-based chemotherapy impairs endothelial function and causes elevation of endothelial and pro-inflammatory acting proteins [C-reactive protein, von Willebrand factor, plasminogen activator inhibitor (PAI-1), and tissue-type plasminogen activator], whereas patients with elevated PAI-1 are in a higher risk of developing metabolic syndrome (126). These pathophysiological changes may result in intima-media thickening, endothelial injury, and dysfunction (127, 128). Animal model studies indicated that cisplatin may exhibit its own cardiotoxic properties by an increase in caspase-3 activity that leads to apoptosis of cardiomyocytes and subsequent drop of cardiac muscle contraction (129). Recently published study pointed out possible arrhythmogenic potential of cisplatin. In large retrospective study patients who received cisplatin had 4 times increased risk of developing atrial fibrillation (130). And in addition, at least three case reports of cisplatin induced bradycardia were reported, in two cases even with a necessary pacemaker implantation for atrioventricular block (131, 132).

More studies are needed to prove arrhythmogenic potential of cisplatin. Although several studies have not confirmed cisplatin-related depression of cardiac systolic function (133, 134), few case reports describing the cardiotoxic potential of cisplatin are documented—a case report of a 27-year-old man presenting with acute anterior myocardial infarction after chemotherapy with cisplatin and a case of a 53-year-old woman whose ejection fraction dropped from 70% to 48% after the third cycle of cisplatin-based chemotherapy. These reports suggest that, in rare cases, cisplatin may cause depression of the systolic function of a left ventricle by the mechanism documented in animal models (135, 136).

### 1.4. Rituximab

Rituximab is a monoclonal anti-CD20 antibody used for the treatment of hematooncological malignancies originating from CD20-expressing B-cells, such as chronic lymphocytic leukemia (CLL), diffuse large B cell lymphoma, follicular lymphoma, or for selected autoimmune diseases (rheumatoid arthritis or systemic lupus erythematosus) (137–139).

Administration of rituximab may potentially lead to arrhythmias, as it was reported in the European phase II study of rituximab. In this study cardiac arrhythmia was detected in 8.3% of patients (140). Observed arrhythmias included supraventricular tachycardia or ventricular tachycardia. The proposed underlying mechanism of rituximab-induced arrhythmias is due to the release of cytokines such as IL-6 and TNF-α. A case report documented rituximab treatment-related release of cytokines, that led to vasoconstriction, platelet activation, and rupture of atherosclerotic plaque in coronary arteries resulting in myocardial infarction (137, 141). Nevertheless, according to a retrospective study of 2,350 patients, therapy with rituximab was not associated with increased occurrence of cardiotoxicity (142).

The incidence of any cardiac event during treatment with CHOP vs. R-CHOP was 35% vs. 47% respectively, however, the incidence of severe cardiac events did not differ between both groups that is in accordance with the assumption of no significant cardiac toxicity of rituximab (143). However, a case report of a 66-year-old man who developed Takotsubo cardiomyopathy after receiving rituximab for CLL, or a case of a 51-year-old man, who developed non-ischemic cardiomyopathy after rituximab intravenous administration for membranous nephropathy, are documented (137, 144). Both cases point out the necessity of close monitoring cardiotoxicity during treatment.

### 1.5. Vinca alkaloids

Vinca alkaloids are anti-cancer drugs that have been widely used since 1963. These include vincristine, vinblastine, and vindesine (145).

Vincristine acts as a mitosis blocker by inhibiting the polymerization of tubulin and incorporation into microtubules, which leads to programmed cell death. Its anti-neoplastic effect is followed by dose-limiting neurotoxicity (145). Cardiac toxicity of vincristine remains unexplored. Interestingly, according to a mice study, vincristine may prevent DOX-induced cardiomyopathy through activation of pro-survival signal mediated through Akt and diminished cytochrome C release (146).

On the contrary, patients on long-term vincristine treatment had a higher incidence of an abnormal global longitudinal strain than controls (147). Moreover, few case reports of adverse cardiovascular events after administration of vincristine such as coronary spasm or vinorelbine-related non-ST elevation acute coronary syndrome were published (148, 149). Vinca alkaloid neurotoxicity may impair the cardiac autonomic nervous system affecting the regulation of heart rate and blood pressure (150).

### 1.6. Bleomycin

Bleomycin belongs to a family of glycopeptide antibiotics with anti-neoplastic activity based on the incorporation of thymidine into deoxyribonucleic acid (DNA). Its main adverse event is pulmonary toxicity (151). Especially in small vessels, bleomycin may be responsible for the endothelial cell injury resulting in the development of Raynaud phenomenon (152). Endothelial toxicity may be mediated via bleomycin-induced E-selectin expression on the endothelial cells that triggers inflammatory response (153).

Clear evidence about the potential cardiovascular toxicity of bleomycin is missing because the majority of studies of cardiotoxic effects include bleomycin in combination with various other antineoplastic agents (154). However, few case reports have been published describing acute chest pain syndrome after bleomycin infusion (155, 156). In 2021 Gozhenko et al. published a study on a rat model in which two weeks after bleomycin administration myocardial weight decreased and repeated administration led to irreversible changes in the myocardium and endothelial dysfunction, which resulted in myocardial infarction. This study proposed a possible link between bleomycin and cardiotoxicity (157).

### 1.7. Dacarbazine

Dacarbazine is a methylating drug used for the treatment of malignant melanoma, sarcoma, or HL (158). Dacarbazine side effects include vomiting, neutropenia, myelosuppression, or alopecia. Any evidence on the cardiotoxic effects of this agent has not yet been reported (159).

### 1.8. Etoposide

Etoposide anti-neoplastic effect is mediated by targeting TOP2 leading to DNA breaks. Etoposide is used for the treatment of lung cancer, lymphoma and leukemia (160, 161).

Although etoposide is not generally recognized as a cardiotoxic drug, associations between etoposide and cardiac damage have been described.

Main cardiac adverse events include myocardial infarction with vasospasm, direct injury to the myocardial wall, or immune system dysregulation as a proposed mechanism (162, 163).

Several case reports showed that treatment with etoposide is associated with arrhythmogenic potential. Current literature describes a case of a 57-year-old man who suffered from a reversible atrial fibrillation episode a few minutes following etoposide infusion or a case of bradycardia and QTc shortly after etoposide infusion (164, 165).

Cardiotoxicity of high-dose etoposide compared to high-dose CYP in patients undergoing stem cell mobilization was assessed according to the levels of NT-proBNP in a study from Ozkan et al. and results showed that high-dose etoposide was 5,25 times more cardiotoxic than CTX (166). On the contrary, etoposide is regularly used in combination with DOX. According to *in vitro* study on cardiomyoblasts, DOX-induced cardiotoxicity was not increased in the presence of etoposide. This result favored DOX and etoposide combination therapy (167).

### 1.9. Procarbazine

Procarbazine is an alkylating anti-neoplastic agent used for the treatment of HL, malignant melanoma, and brain tumors in children (168). No evidence has yet been published regarding procarbazine cardiotoxicity.

### 1.10. Prednisone

Prednisone acts as an immune-mediating agent and has wide use in therapeutic protocols in hematopoietic malignancies originating from lymphopoiesis (NHL, ALL). Glucocorticoids possess direct and indirect effects on the heart and cell signaling pathways and are essential for normal cardiac function at physiological levels (169). Adrenalectomy in mice leads to deficit in left ventricular function and ECG abnormalities and primary adrenal insufficiency may even lead to cardiogenic shock (169, 170).

Nevertheless, high-dose intravenous corticosteroids were associated with a higher incidence of atrial fibrillation, ventricular tachycardia and bradycardia events (171). Sinus bradycardia was not only described after high intravenous or oral doses, but cases have been reported where sinus bradycardia developed after daily 40 mg oral prednisone (172–175). The underlying mechanism of cardiac arrhythmias remains unclear. Proposed mechanisms include suppression of the cytokine production, modification of the function of the sympathetic nervous system, or through alteration of potassium flux across the cell membrane (176, 177).

### 1.11. Thoracic irradiation

Thoracic irradiation may cause direct damage to any part of the heart and most commonly affects valves, followed by coronary arteries, myocardium, heart conduction system and pericardium (178). The mean irradiation dose to the heart is significantly lower using involved node radiotherapy than the mantle field technique (179).

#### 1.11.1. Valvular dysfunction

Valvular susceptibility is higher in the left ventricle (predominantly on the aortic valve) due to higher pressure. Irradiation-induced aortic regurgitation is more common than stenosis. This is in contrast with myocardial damage and fibrosis, that are more frequently manifest in the right heart compartments, possibly due to the position of the right ventricle and anterior radiation fields (178, 180, 181).

Severe valvular disease during a median follow-up of 13 years was diagnosed in 24,5% of HL survivors with mediastinal radiotherapy vs. 3,4% without mediastinal radiotherapy. Up to 18% of monitored patients were indicated to have valvular surgery in the group which received mediastinal irradiation vs. none in the group without mediastinal radiation (182). Irradiation induces degenerative changes and the absence of valvular disease early after radiation does not indicate a low risk of late-onset valvular disease (decades after therapy discontinuation) (183).

#### 1.11.2. Coronary artery involvement

Significant coronary artery disease developed in 18% of patients in 10 years follow-up after radiotherapy in the study by Horimoto et al. (180). Radiotherapy in patients with HL leads to the formation of macrophage-rich inflammatory atherosclerotic lesions with a tendency to intraplaque hemorrhage. This pathophysiological process directly increases the risk of atherosclerotic events (184). Radiation-associated coronary artery disease (CAD) predominantly manifests in proximal parts of the left main and right ostial coronary arteries due to higher radiation doses to the anterior surface of the heart and often affects women with low-risk factors for CAD (185, 186). Manifestation of CAD is further promoted by radiotherapy-induced chronic inflammatory state and higher prevalence of diabetes (or metabolic syndrome) (125, 187, 188). The risk of CAD manifestation was directly proportional to the mean heart dose, however, according to the study of Maazen et al., an increased level of physical activity decreased the risk of coronary artery disease in a similar manner to the general population (189).

#### 1.11.3. Myocardial damage and conduction abnormalities

Main adverse effects of irradiation result in microcirculatory damage and altered collagen concentration. These changes may be responsible for defective diastolic distensibility of the ventricles and marked fibrosis may cause arrhythmia such as AV block (190, 191). Defective diastolic distensibility may lead to restrictive cardiomyopathy and cause CHF which can be even more potentiated by radiation-induced coronary artery disease, pericardial or valvular disease. Moreover, case reports of takotsubo cardiomyopathy following chest radiation have been reported (192, 193).

#### 1.11.4. Pericardium involvement

Extensive fibrous thickening and excessive pericardial fluid were predominantly associated with older radiotherapy techniques (44). Acute pericarditis often presents in the first year after irradiation (180). Acute pericarditis results from small blood vessels proliferation through the pericardium. These vessels are usually damaged, resulting in increased ischemia and fibrosis. Furthermore, venous and lymphatic vessel damage impairs the ability to drain extracellular fluid (44). Thickening of pericardium was diagnosed in 15% of patients treated with older radiation techniques for HL with a median radiation dose of 41 Gy in a study by Lund et al. (164)

In another study by Galper et al. pericardial disease was identified in 9 patients out of 1,279 HL survivors who were treated with median dose of 40 Gy and cumulatively only 1,3% of patients needed pericardial surgery after 25 years of follow up (181, 194). Severe damage of pericardium seems to be a rare complication in standard radiation doses.

Thoracic irradiation in childhood cancer survivors is an emerging issue with a respect to the years of survival after therapy discontinuation. According to the study by Mulrooney et al. cardiac radiation exposure of 15 Gy or more during childhood increased the relative hazard of cardiac events (CHF, myocardial infarction, pericardial disease, and valvular abnormalities) by twofold to sixfold compared to non-irradiated long-term survivors (24).

In a large-scale study of 13,060 childhood cancer survivors who were followed up to the age of 50 years an individual prediction model of ischemic heart disease was proposed, and cancer survivors were categorized according to sex, chemotherapy, and heart-absorbed radiation dose to low-risk vs. high-risk groups. The cumulative incidence of ischemic heart disease at the age of 50 years among low-risk survivors was <5% compared with 20% for high-risk groups (cumulative incidence for siblings were 1%) (195). The cumulative incidence of cardiac disease in HL survivors decreases from 21% (after mediastinal radiation dose of 36 Gy) to 10%, 6%, 5%, 3% after lower mediastinal radiation doses (30 Gy, 25 Gy, 20 Gy, 0 Gy) (178).

Currently, novel radiation techniques such as mantle field techniques are employed and high doses to anterior parts of the heart are partly compensated by boost treatment from non-anterior angles potentially decreasing the risk of cardiotoxicity (196).

Prevention is mainly focused on using modern irradiation techniques with reducing heart's exposure to radiation such as prone positioning, deep inspiratory breath holding, intensity modulated techniques, intraoperative irradiation (197). Yet, there is no medicament to reduce radiation damage but in rat models N-acetyl cysteine ameliorated cardiac injury induced by RT and hence possibly could be used as radioprotector (198).

### 1.12. Novel specific targeted therapy

#### 1.12.1. Bruton's tyrosine kinase inhibitor

Assumingly, new cancer therapy-related cardiovascular complications will be discovered with advances in targeted therapy for malignant lymphoma (199). Ibrutinib (Bruton's tyrosine kinase (BTK) inhibitor nowadays used for refractory chronic lymphocytic leukemia or mantle cell lymphoma (200) increases the risk of atrial fibrillation potentially via inhibition of cardiac PI3K-Akt signaling (201). The second generation BTK inhibitors were introduced to reduce BTK-related cardiotoxicity. In this generation, adverse effect occur in 6.3% vs. 20.8% cases in first generation (202). Although according to Arustamyan et al. (202), the second generation BTK inhibitors have increased incidence of other adverse effects such as endocrine, gastrointestinal, neurological etc. Pirtobrutinib is highly selective BTK inhibitor and is classified as third generation. Mato et al. (203) found that less than 1% of patients suffered from atrial fibrillation and flutter and possibly unrelated to Pirtobrutinib.

#### 1.12.2. Immune checkpoint inhibitors (ICI)

Immune checkpoint inhibitors (ICI) fight tumor cells dominantly by the activation of T cells (204). Certain ICI posses significant efficacy with an overall response rate in relapse/refractory HL nevertheless in rare cases the cardiotoxic adverse effect may be lethal (205). ICI associated cardiotoxicity is mainly represented by myocarditis, pericarditis, arrhythmias and rarely by coronary spasm (206–208). The exact mechanism of ICI-related cardiotoxicity remains unclear, however proposed mechanisms include shared antigen between tumor and myocardium, T-cell receptor targeting homologous muscle antigen or certain T-cell receptors targeting dissimilar antigens (209). ICI-related cardiotoxicity in solid tumors therapy was described in 7% of patients with diabetes mellitus as an independent risk factor (204). ICI possesses not only acute cardiotoxicity but also late-onset cardiotoxicity. In the study from Dolladille (200), late-onset cardiotoxicity was defined as any cardiac adverse effect observed after more than 90 days following therapy initiation. These adverse effects were mainly represented by heart failure emphasizing the necessity of prolonged cardiac follow-up after ICI therapy (200).

#### 1.12.3. Chimeric antigen receptor T (CAR-T) cells

Immune therapy with CAR-T cells is currently reserved for patients suffering from late stages or refractory hematological malignancies. This novel therapeutic approach yields promising results, however its alteration of immune response leads to various cytokine-related adverse events (210). The incidence of cytokine release syndrome (CRS), resulting from overactivation of immune system, occurs in 70%–90% patients receiving CAR-T cell therapy. Approximately one third of all patients with CRS experience any cardiac adverse events (211, 212). Most common are tachyarrhytmias (both supraventricular and ventricular), QT-interval prolongation, myocardial ischemia and thromboembolism (213). Further investigation of exact mechansims of CAR-T cell therapy-related cardiotoxicity is necessary to discover potential targets of cardioprotective medication.

## 2. Discussion and future perspectives

In this review, we revisited the topic of cardiotoxicity of conventional chemotherapeutics that still remain a mainstay of therapeutic protocols used to treat malignant lymphomas. Moreover it is still important to note, that many patients treated in young age, that we follow-up today in cardiology/oncology departments, received treatment based on chemotherapy only and still need to be monitored for cardiotoxicity. An update to “old and known” drugs pharmacology is important as novel pharmacokinetic and pharmacodynamic properties are steadily being discovered. Typical example is the pathophysiology underlying TOP2 isoform-mediated cytotoxic effect of DOX. The knowledge of tumor expressing predominantly TOP2*α*, while other tissues expressing mainly TOP2β, rise a potential for developing drugs of the anthracycline class selectively targeting TOP2α. These may lead to reduction of cardiac damage. Moreover, based on the individual expression of TOP2β, a suitable (or alternative) chemotherapy regimen may be chosen, however further studies are needed to explore this concept (51).

Current experimental studies on possible cardioprotective agents have mostly been conducted on animal (murine) model with promising results. However, large-scale human clinical trials failed to provide significant results. One example of a successful implementation of cardioprotective agent in clinical practice is dexrazoxane. Several clinical trials on cardioprotection during anti-cancer treatment are currently conducted including STOP-CA with atorvastatin/DOX (214). Early results of STOP-CA presented by Neilan TG at ACC/WCC 2023 show that patients receiving 40 mg of oral atorvastatin were less likely to develop 10% or greater decline in LVEF than those receiving placebo. Female individuals over the age of 52, obese patients and those receiving doses of anthracyclines greater than 250 mg/m^2^ benefited the most from daily atorvastatin (215). Clinical trial evaluating the effects of ACE-i and β-blockers in management of cardiotoxicity in cancer patients are expected to be completed in 2030 (216).

Another approach to reduce impact of anti-cancer chemotherapy on cardiac function is to explore cardioprotective effect of medication commonly used to treat cardiac disorders in non-cancer patients (e.g., ischemic heart disease, congestive heart failure etc.). As Cardinale et al. (87) reported, main benefit of ACE-i and BB therapy in DOX-treated patients comes from its early initiation when a drop of LVEF is discovered. This implicates the necessity of proper follow-up visits as reported in ESC 2022 cardiooncology guidelines (6).

As novel target/immune therapeutics are steadily introduced to clinical practice, close cardiotoxicity monitoring is essential. Although this treatment is marked as “targeted” (focusing its toxic properties on tumor tissue), clinical trials still reveal significant negative effects on cardiovascular system. Of the most pronounced complications of ICI therapy, cardiotoxicity may result from shared antigen or triggered systemic immune response and is currently managed by immunesuppresants. These, however, may modulate both cardiac toxicity and undesirably anti-tumor effect (217). Thus ICI treatment adverse events pose a future therapeutic challenge.

Similar complications rise from CAR-T cells-related overactivation of immune system, CRS, which is classified as on-target, on-tumor toxicity (218). Preventing the progression of CRS impacts overall cardiovascular outcomes in CAR-T cell patients. This can currently be achieved non-specifically by corticosteroids (potentially causing cardiac adverse events themselves and affecting anti-tumor effect of CAR-T cells) or more specifically by Tocilizumab, an anti-IL6 monoclonal antibody (217). Tocilizumab seems to be a promising cardioprotective agent used to manage CRS with less effect on CAR-T anti-tumor action (219).

Antigenic similarity remains an issue of CAR-T cell therapy. Tumor antigens may resemble peptide sequences of cardiac cell proteins as described in recent studies (220). These toxicities are referred to as on-target, off-tumour toxicities and are generally managed by immune modulatory drugs or by methods of controlling CAR-T cells activity. These have mostly been described in therapy regimens for solid tumors and are relatively rare in the literature (220–222). However, these complications need to be closely monitored and overcome, possibly by modern technologies, such as logic-gating circuits and synthetic biology approaches (223).

Regarding BTK inhibitors, highly selective third generation is expected to balance positives of the first- and second- generation drugs of this class (203). Particularly Pirtobrutinib was found to be safe and efficacious in multiple malignancies in clinical setting (203) and was recently approved for the treatment of adult patients with relapsed or refractory mantle cell lymphoma (224).

## 3. Conclusion

Cardiotoxicity of anti-cancer treatment in malignant lymphoma remains a challenge for oncologists and cardiologists. In this article, we reviewed mechanisms of specific cardiotoxic properties of currently used anti-cancer drugs for treatment of malignant lymphoma and their clinical manifestations (*in summary, see* Table 1). These statements imply the need for further research on management of chemotherapy-related cardiac toxicity.

In 2022, European Society of Cardiology published new cardiooncology guidelines with detailed risk stratification of patients undergoing chemotherapy and follow-up strategy during and after chemotherapy cessation (6). Close collaboration of cardiologists and oncologists is essential to provide appropriate care. Hopefully new and/or running clinical trials will come up with new effective therapeutic approaches including specific cardioprotective targeted therapy that will reduce cardiotoxicity burden of currently used anti-cancer treatment.

## References

[1] Swerdlow SH Campo E Pileri SA Harris NL Stein H Siebert R The 2016 revision of the World Health Organization classification of lymphoid neoplasms. Blood. (2016) 127(20):2375–90. 10.1182/blood-2016-01-643569 26980727 PMC4874220

[2] Shirakawa S Kobayashi T Kita K Ohno T Miwa H. [Malignant lymphoma]. Gan to Kagaku Ryoho. (1989) 16(4 Pt 2-1):951–8. 2730035

[3] Villacampa G Dienstmann R Bosch F Abrisqueta P. Combination of novel molecular targeted agent plus R-CHOP-based regimen versus R-CHOP alone in previously untreated diffuse large B-cell lymphoma (DLBCL) patients: a systematic review and meta-analysis. Ann Hematol. (2021) 100(12):2969–78. 10.1007/s00277-021-04623-8 34378095

[4] Gallamini A Tarella C Viviani S Rossi A Patti C Mulé A Early chemotherapy intensification with escalated BEACOPP in patients with advanced-stage hodgkin lymphoma with a positive interim positron emission tomography/computed tomography scan after two ABVD cycles: long-term results of the GITIL/FIL HD 0607 trial. J Clin Oncol. (2018) 36(5):454–62. 10.1200/JCO.2017.75.2543 29360414

[5] Diehl V Franklin J Hasenclever D Tesch H Pfreundschuh M Lathan B BEACOPP, a new dose-escalated and accelerated regimen, is at least as effective as COPP/ABVD in patients with advanced-stage hodgkin’s lymphoma: interim report from a trial of the German Hodgkin’s Lymphoma Study Group. J Clin Oncol. (1998) 16(12):3810–21. 10.1200/JCO.1998.16.12.3810 9850026

[6] Lyon AR López-Fernández T Couch LS Asteggiano R Aznar MC Bergler-Klein J 2022 ESC guidelines on cardio-oncology developed in collaboration with the European hematology association (EHA), the European society for therapeutic radiology and oncology (ESTRO) and the international cardio-oncology society (IC-OS). Eur Heart J. (2022) 43(41):4229–361. 10.1093/eurheartj/ehac244 36017568

[7] Boyne DJ Mickle AT Brenner DR Friedenreich CM Cheung WY Tang KL Long-term risk of cardiovascular mortality in lymphoma survivors: a systematic review and meta-analysis. Cancer Med. (2018) 7(9):4801–13. 10.1002/cam4.1572 30112841 PMC6143935

[8] van Nimwegen FA Schaapveld M Janus CP Krol AD Petersen EJ Raemaekers JM Cardiovascular disease after Hodgkin lymphoma treatment: 40-year disease risk. JAMA Intern Med. (2015) 175(6):1007–17. 10.1001/jamainternmed.2015.1180 25915855

[9] Jain D Russell RR Schwartz RG Panjrath GS Aronow W. Cardiac complications of cancer therapy: pathophysiology, identification, prevention, treatment, and future directions. Curr Cardiol Rep. (2017) 19(5):36. 10.1007/s11886-017-0846-x 28374177

[10] Di Marco A Cassinelli G Arcamone F. The discovery of daunorubicin. Cancer Treat Rep. (1981) 65(Suppl 4):3–8. 7049379

[11] Di Marco A Gaetani M Scarpinato B. Adriamycin (NSC-123,127): a new antibiotic with antitumor activity. Cancer Chemother Rep. (1969) 53(1):33–7. 5772652

[12] Volkova M Russell R. Anthracycline cardiotoxicity: prevalence, pathogenesis and treatment. Curr Cardiol Rev. (2011) 7(4):214–20. 10.2174/157340311799960645 22758622 PMC3322439

[13] Rigacci L Annibali O Kovalchuk S Bonifacio E Pregnolato F Angrilli F Nonpeghylated liposomal doxorubicin combination regimen (R-COMP) for the treatment of lymphoma patients with advanced age or cardiac comorbidity. Hematol Oncol. (2020) 38(4):478–86. 10.1002/hon.2764 32542788 PMC7689940

[14] Minotti G Menna P Salvatorelli E Cairo G Gianni L. Anthracyclines: molecular advances and pharmacologic developments in antitumor activity and cardiotoxicity. Pharmacol Rev. (2004) 56(2):185–229. 10.1124/pr.56.2.6 15169927

[15] Von Hoff DD Layard MW Basa P Davis HL Von Hoff AL Rozencweig M Risk factors for doxorubicin-induced congestive heart failure. Ann Intern Med. (1979) 91(5):710–7. 10.7326/0003-4819-91-5-710 496103

[16] Hayek ER Speakman E Rehmus E. Acute doxorubicin cardiotoxicity. N Engl J Med. (2005) 352(23):2456–7. 10.1056/NEJM200506093522321 15944435

[17] Herrmann J. Adverse cardiac effects of cancer therapies: cardiotoxicity and arrhythmia. Nat Rev Cardiol. (2020) 17(8):474–502. 10.1038/s41569-020-0348-1 32231332 PMC8782611

[18] Ben Aharon I Bar Joseph H Tzabari M Shenkman B Farzam N Levi M Doxorubicin-induced vascular toxicity--targeting potential pathways may reduce procoagulant activity. PLoS One. (2013) 8(9):e75157. 10.1371/journal.pone.0075157 24073244 PMC3779248

[19] Arbel Y Swartzon M Justo D. QT Prolongation and torsades de pointes in patients previously treated with anthracyclines. Anticancer Drugs. (2007) 18(4):493–8. 10.1097/CAD.0b013e328012d023 17351403

[20] Voit J Tibrewala A Akhter N. Heart of the matter: reverse takotsubo syndrome in an anthracycline-exposed oncology patient. BMJ Case Rep. (2018) 2018:bcr-2018-226378. 10.1136/bcr-2018-226378 30287630 PMC6194443

[21] Mubarak G Haddadin M Samra B Luhrs C Taiwo E. Doxorubicin-associated takotsubo cardiomyopathy in a patient with adult T-cell leukemia/lymphoma. Clin Case Rep. (2019) 7(12):2466–71. 10.1002/ccr3.2504 31893081 PMC6935604

[22] Swain SM Whaley FS Ewer MS. Congestive heart failure in patients treated with doxorubicin: a retrospective analysis of three trials. Cancer. (2003) 97(11):2869–79. 10.1002/cncr.11407 12767102

[23] Jensen BV Skovsgaard T Nielsen SL. Functional monitoring of anthracycline cardiotoxicity: a prospective, blinded, long-term observational study of outcome in 120 patients. Ann Oncol. (2002) 13(5):699–709. 10.1093/annonc/mdf132 12075737

[24] Mulrooney DA Yeazel MW Kawashima T Mertens AC Mitby P Stovall M Cardiac outcomes in a cohort of adult survivors of childhood and adolescent cancer: retrospective analysis of the childhood cancer survivor study cohort. Br Med J. (2009) 339:b4606. 10.1136/bmj.b4606 19996459 PMC3266843

[25] van Dalen EC van der Pal HJ Kok WE Caron HN Kremer LC. Clinical heart failure in a cohort of children treated with anthracyclines: a long-term follow-up study. Eur J Cancer. (2006) 42(18):3191–8. 10.1016/j.ejca.2006.08.005 16987655

[26] Youssef G Links M. The prevention and management of cardiovascular complications of chemotherapy in patients with cancer. Am J Cardiovasc Drugs. (2005) 5(4):233–43. 10.2165/00129784-200505040-00003 15984906

[27] Wouters KA Kremer LC Miller TL Herman EH Lipshultz SE. Protecting against anthracycline-induced myocardial damage: a review of the most promising strategies. Br J Haematol. (2005) 131(5):561–78. 10.1111/j.1365-2141.2005.05759.x 16351632

[28] Deng S Wojnowski L. Genotyping the risk of anthracycline-induced cardiotoxicity. Cardiovasc Toxicol. (2007) 7(2):129–34. 10.1007/s12012-007-0024-2 17652817

[29] Floyd JD Nguyen DT Lobins RL Bashir Q Doll DC Perry MC. Cardiotoxicity of cancer therapy. J Clin Oncol. (2005) 23(30):7685–96. 10.1200/JCO.2005.08.789 16234530

[30] Villani F Galimberti M Zunino F Monti E Rozza A Lanza E Prevention of doxorubicin-induced cardiomyopathy by reduced glutathione. Cancer Chemother Pharmacol. (1991) 28(5):365–9. 10.1007/BF00685691 1914080

[31] Radi R Turrens JF Chang LY Bush KM Crapo JD Freeman BA. Detection of catalase in rat heart mitochondria. J Biol Chem. (1991) 266(32):22028–34. 10.1016/S0021-9258(18)54740-2 1657986

[32] Senkus E Jassem J. Cardiovascular effects of systemic cancer treatment. Cancer Treat Rev. (2011) 37(4):300–11. 10.1016/j.ctrv.2010.11.001 21126826

[33] Scully RE Lipshultz SE. Anthracycline cardiotoxicity in long-term survivors of childhood cancer. Cardiovasc Toxicol. (2007) 7(2):122–8. 10.1007/s12012-007-0006-4 17652816

[34] Davies KJ Doroshow JH. Redox cycling of anthracyclines by cardiac mitochondria. I. Anthracycline radical formation by NADH dehydrogenase. J Biol Chem. (1986) 261(7):3060–7. 10.1016/S0021-9258(17)35746-0 3456345

[35] Kang YJ Zhou ZX Wang GW Buridi A Klein JB. Suppression by metallothionein of doxorubicin-induced cardiomyocyte apoptosis through inhibition of p38 mitogen-activated protein kinases. J Biol Chem. (2000) 275(18):13690–8. 10.1074/jbc.275.18.13690 10788488

[36] Slørdal L Spigset O. Heart failure induced by non-cardiac drugs. Drug Saf. (2006) 29(7):567–86. 10.2165/00002018-200629070-00003 16808550

[37] Chua CC Liu X Gao J Hamdy RC Chua BH. Multiple actions of pifithrin-alpha on doxorubicin-induced apoptosis in rat myoblastic H9c2 cells. Am J Physiol Heart Circ Physiol. (2006) 290(6):H2606–13. 10.1152/ajpheart.01138.2005 16687611

[38] Chen B Peng X Pentassuglia L Lim CC Sawyer DB. Molecular and cellular mechanisms of anthracycline cardiotoxicity. Cardiovasc Toxicol. (2007) 7(2):114–21. 10.1007/s12012-007-0005-5 17652815

[39] Childs AC Phaneuf SL Dirks AJ Phillips T Leeuwenburgh C. Doxorubicin treatment in vivo causes cytochrome C release and cardiomyocyte apoptosis, as well as increased mitochondrial efficiency, superoxide dismutase activity, and Bcl-2:Bax ratio. Cancer Res. (2002) 62(16):4592–8. 12183413

[40] Lebrecht D Walker UA. Role of mtDNA lesions in anthracycline cardiotoxicity. Cardiovasc Toxicol. (2007) 7(2):108–13. 10.1007/s12012-007-0009-1 17652814

[41] Binaschi M Zunino F Capranico G. Mechanism of action of DNA topoisomerase inhibitors. Stem Cells. (1995) 13(4):369–79. 10.1002/stem.5530130408 7549896

[42] Lawrence JW Darkin-Rattray S Xie F Neims AH Rowe TC. 4-Quinolones Cause a selective loss of mitochondrial DNA from mouse L1210 leukemia cells. J Cell Biochem. (1993) 51(2):165–74. 10.1002/jcb.240510208 8440750

[43] Eidenschink AB Schröter G Müller-Weihrich S Stern H. Myocardial high-energy phosphate metabolism is altered after treatment with anthracycline in childhood. Cardiol Young. (2000) 10(6):610–7. 10.1017/S1047951100008891 11117394

[44] Lipshultz SE Adams MJ Colan SD Constine LS Herman EH Hsu DT Long-term cardiovascular toxicity in children, adolescents, and young adults who receive cancer therapy: pathophysiology, course, monitoring, management, prevention, and research directions: a scientific statement from the American heart association. Circulation. (2013) 128(17):1927–95. 10.1161/CIR.0b013e3182a88099 24081971

[45] Vejpongsa P Yeh ET. Prevention of anthracycline-induced cardiotoxicity: challenges and opportunities. J Am Coll Cardiol. (2014) 64(9):938–45. 10.1016/j.jacc.2014.06.1167 25169180

[46] Martin OJ Lai L Soundarapandian MM Leone TC Zorzano A Keller MP A role for peroxisome proliferator-activated receptor *γ* coactivator-1 in the control of mitochondrial dynamics during postnatal cardiac growth. Circ Res. (2014) 114(4):626–36. 10.1161/CIRCRESAHA.114.302562 24366168 PMC4061768

[47] Sihag S Cresci S Li AY Sucharov CC Lehman JJ. PGC-1alpha and ERRalpha target gene downregulation is a signature of the failing human heart. J Mol Cell Cardiol. (2009) 46(2):201–12. 10.1016/j.yjmcc.2008.10.025 19061896 PMC2681265

[48] Zhang S Liu X Bawa-Khalfe T Lu LS Lyu YL Liu LF Identification of the molecular basis of doxorubicin-induced cardiotoxicity. Nat Med. (2012) 18(11):1639–42. 10.1038/nm.2919 23104132

[49] Finkel T. Cell biology: a clean energy programme. Nature. (2006) 444(7116):151–2. 10.1038/444151a 17093435

[50] Vejpongsa P Yeh ET. Topoisomerase 2β: a promising molecular target for primary prevention of anthracycline-induced cardiotoxicity. Clin Pharmacol Ther. (2014) 95(1):45–52. 10.1038/clpt.2013.201 24091715

[51] Kersting G Tzvetkov MV Huse K Kulle B Hafner V Brockmöller J Topoisomerase II beta expression level correlates with doxorubicin-induced apoptosis in peripheral blood cells. Naunyn Schmiedebergs Arch Pharmacol. (2006) 374(1):21–30. 10.1007/s00210-006-0091-0 16957942

[52] Jordan JH Castellino SM Meléndez GC Klepin HD Ellis LR Lamar Z Left ventricular mass change after anthracycline chemotherapy. Circ Heart Fail. (2018) 11(7):e004560. 10.1161/CIRCHEARTFAILURE.117.004560 29991488 PMC6729136

[53] Ferreira de Souza T Quinaglia A C Silva T Osorio Costa F Shah R Neilan TG Velloso L Anthracycline therapy is associated with cardiomyocyte atrophy and preclinical manifestations of heart disease. JACC Cardiovasc Imaging. (2018) 11(8):1045–55. 10.1016/j.jcmg.2018.05.012 30092965 PMC6196358

[54] Willis MS Rojas M Li L Selzman CH Tang RH Stansfield WE Muscle ring finger 1 mediates cardiac atrophy in vivo. Am J Physiol Heart Circ Physiol. (2009) 296(4):H997–H1006. 10.1152/ajpheart.00660.2008 19168726 PMC2670686

[55] Willis MS Parry TL Brown DI Mota RI Huang W Beak JY Doxorubicin exposure causes subacute cardiac atrophy dependent on the striated muscle-specific ubiquitin ligase MuRF1. Circ Heart Fail. (2019) 12(3):e005234. 10.1161/CIRCHEARTFAILURE.118.005234 30871347 PMC6422170

[56] Moghe A Ghare S Lamoreau B Mohammad M Barve S McClain C Molecular mechanisms of acrolein toxicity: relevance to human disease. Toxicol Sci. (2015) 143(2):242–55. 10.1093/toxsci/kfu233 25628402 PMC4306719

[57] Rom O Kaisari S Aizenbud D Reznick AZ. The effects of acetaldehyde and acrolein on muscle catabolism in C2 myotubes. Free Radic Biol Med. (2013) 65:190–200. 10.1016/j.freeradbiomed.2013.06.024 23792774

[58] Huang C Zhang X Ramil JM Rikka S Kim L Lee Y Juvenile exposure to anthracyclines impairs cardiac progenitor cell function and vascularization resulting in greater susceptibility to stress-induced myocardial injury in adult mice. Circulation. (2010) 121(5):675–83. 10.1161/CIRCULATIONAHA.109.902221 20100968 PMC2834271

[59] Gianni L Herman EH Lipshultz SE Minotti G Sarvazyan N Sawyer DB. Anthracycline cardiotoxicity: from bench to bedside. J Clin Oncol. (2008) 26(22):3777–84. 10.1200/JCO.2007.14.9401 18669466 PMC3018290

[60] Takemura G Fujiwara H. Doxorubicin-induced cardiomyopathy from the cardiotoxic mechanisms to management. Prog Cardiovasc Dis. (2007) 49(5):330–52. 10.1016/j.pcad.2006.10.002 17329180

[61] Spallarossa P Garibaldi S Altieri P Fabbi P Manca V Nasti S Carvedilol prevents doxorubicin-induced free radical release and apoptosis in cardiomyocytes in vitro. J Mol Cell Cardiol. (2004) 37(4):837–46. 10.1016/j.yjmcc.2004.05.024 15380674

[62] Dowd NP Scully M Adderley SR Cunningham AJ Fitzgerald DJ. Inhibition of cyclooxygenase-2 aggravates doxorubicin-mediated cardiac injury in vivo. J Clin Invest. (2001) 108(4):585–90. 10.1172/JCI200111334 11518732 PMC209394

[63] Kotamraju S Chitambar CR Kalivendi SV Joseph J Kalyanaraman B. Transferrin receptor-dependent iron uptake is responsible for doxorubicin-mediated apoptosis in endothelial cells: role of oxidant-induced iron signaling in apoptosis. J Biol Chem. (2002) 277(19):17179–87. 10.1074/jbc.M111604200 11856741

[64] Matsui T Tao J del Monte F Lee KH Li L Picard M Akt activation preserves cardiac function and prevents injury after transient cardiac ischemia in vivo. Circulation. (2001) 104(3):330–5. 10.1161/01.CIR.104.3.330 11457753

[65] Taniyama Y Walsh K. Elevated myocardial Akt signaling ameliorates doxorubicin-induced congestive heart failure and promotes heart growth. J Mol Cell Cardiol. (2002) 34(10):1241–7. 10.1006/jmcc.2002.2068 12392981

[66] Herzog W. The multiple roles of titin in muscle contraction and force production. Biophys Rev. (2018) 10(4):1187–99. 10.1007/s12551-017-0395-y 29353351 PMC6082311

[67] Lou H Danelisen I Singal PK. Involvement of mitogen-activated protein kinases in adriamycin-induced cardiomyopathy. Am J Physiol Heart Circ Physiol. (2005) 288(4):H1925–30. 10.1152/ajpheart.01054.2004 15772336

[68] van Dalen EC Caron HN Dickinson HO Kremer LC. Cardioprotective interventions for cancer patients receiving anthracyclines. Cochrane Database Syst Rev. (2011) 6:CD003917. 10.1002/14651858.CD003917.pub4 PMC6457676 21678342

[69] Georgakopoulos P Roussou P Matsakas E Karavidas A Anagnostopoulos N Marinakis T Cardioprotective effect of metoprolol and enalapril in doxorubicin-treated lymphoma patients: a prospective, parallel-group, randomized, controlled study with 36-month follow-up. Am J Hematol. (2010) 85(11):894–6. 10.1002/ajh.21840 20872550

[70] Gulati G Heck SL Ree AH Hoffmann P Schulz-Menger J Fagerland MW Prevention of cardiac dysfunction during adjuvant breast cancer therapy (PRADA): a 2 × 2 factorial, randomized, placebo-controlled, double-blind clinical trial of candesartan and metoprolol. Eur Heart J. (2016) 37(21):1671–80. 10.1093/eurheartj/ehw022 26903532 PMC4887703

[71] Meattini I Curigliano G Terziani F Becherini C Airoldi M Allegrini G SAFE Trial: an ongoing randomized clinical study to assess the role of cardiotoxicity prevention in breast cancer patients treated with anthracyclines with or without trastuzumab. Med Oncol. (2017) 34(5):75. 10.1007/s12032-017-0938-x 28364270

[72] Elfadadny A Ragab RF Hamada R Al Jaouni SK Fu J Mousa SA Natural bioactive compounds-doxorubicin combinations targeting topoisomerase II-alpha: anticancer efficacy and safety. Toxicol Appl Pharmacol. (2023) 461:116405. 10.1016/j.taap.2023.116405 36716865

[73] Swain SM Whaley FS Gerber MC Ewer MS Bianchine JR Gams RA. Delayed administration of dexrazoxane provides cardioprotection for patients with advanced breast cancer treated with doxorubicin-containing therapy. J Clin Oncol. (1997) 15(4):1333–40. 10.1200/JCO.1997.15.4.1333 9193324

[74] Lipshultz SE Rifai N Dalton VM Levy DE Silverman LB Lipsitz SR The effect of dexrazoxane on myocardial injury in doxorubicin-treated children with acute lymphoblastic leukemia. N Engl J Med. (2004) 351(2):145–53. 10.1056/NEJMoa035153 15247354

[75] Hasinoff BB Patel D Wu X. The oral iron chelator ICL670A (deferasirox) does not protect myocytes against doxorubicin. Free Radic Biol Med. (2003) 35(11):1469–79. 10.1016/j.freeradbiomed.2003.08.005 14642395

[76] Lyu YL Kerrigan JE Lin CP Azarova AM Tsai YC Ban Y Topoisomerase IIbeta mediated DNA double-strand breaks: implications in doxorubicin cardiotoxicity and prevention by dexrazoxane. Cancer Res. (2007) 67(18):8839–46. 10.1158/0008-5472.CAN-07-1649 17875725

[77] Seymour L Bramwell V Moran LA. Use of dexrazoxane as a cardioprotectant in patients receiving doxorubicin or epirubicin chemotherapy for the treatment of cancer. The provincial systemic treatment disease site group. Cancer Prev Control. (1999) 3(2):145–59. 10474762

[78] Marty M Espié M Llombart A Monnier A Rapoport BL Stahalova V Multicenter randomized phase III study of the cardioprotective effect of dexrazoxane (cardioxane) in advanced/metastatic breast cancer patients treated with anthracycline-based chemotherapy. Ann Oncol. (2006) 17(4):614–22. 10.1093/annonc/mdj134 16423847

[79] Gabizon AA. Stealth liposomes and tumor targeting: one step further in the quest for the magic bullet. Clin Cancer Res. (2001) 7(2):223–5. 11234871

[80] McSweeney MD Price LSL Wessler T Ciociola EC Herity LB Piscitelli JA Overcoming anti-PEG antibody mediated accelerated blood clearance of PEGylated liposomes by pre-infusion with high molecular weight free PEG. J Control Release. (2019) 51:311–312:138–46. 10.1016/j.jconrel.2019.08.017 PMC6874909 31454530

[81] O'Brien ME Wigler N Inbar M Rosso R Grischke E Santoro A Reduced cardiotoxicity and comparable efficacy in a phase III trial of pegylated liposomal doxorubicin HCl (CAELYX/Doxil) versus conventional doxorubicin for first-line treatment of metastatic breast cancer. Ann Oncol. (2004) 15(3):440–9. 10.1093/annonc/mdh097 14998846

[82] Cardinale D Sandri MT Martinoni A Tricca A Civelli M Lamantia G Left ventricular dysfunction predicted by early troponin I release after high-dose chemotherapy. J Am Coll Cardiol. (2000) 36(2):517–22. 10.1016/S0735-1097(00)00748-8 10933366

[83] Vasatova M Pudil R Horacek JM Buchler T. Current applications of cardiac troponin T for the diagnosis of myocardial damage. Adv Clin Chem. (2013) 61:33–65. 10.1016/B978-0-12-407680-8.00002-6 24015599

[84] Sandri MT Salvatici M Cardinale D Zorzino L Passerini R Lentati P N-terminal pro-B-type natriuretic peptide after high-dose chemotherapy: a marker predictive of cardiac dysfunction? Clin Chem. (2005) 51(8):1405–10. 10.1373/clinchem.2005.050153 15932966

[85] Mihalcea D Memis H Balinisteanu A Vladareanu AM Mihaila S Vinereanu D. Myocardial work-A new tool for early detection of rituximab, cyclophosphamide, doxorubicin, vincristine, prednisone chemotherapy induced-cardiotoxicity in hematological patients. J Clin Ultrasound. (2023) 51(3):377–84. 10.1002/jcu.23388 36331055

[86] Plana JC Galderisi M Barac A Ewer MS Ky B Scherrer-Crosbie M Expert consensus for multimodality imaging evaluation of adult patients during and after cancer therapy: a report from the American society of echocardiography and the European association of cardiovascular imaging. J Am Soc Echocardiogr. (2014) 27(9):911–39. 10.1016/j.echo.2014.07.012 25172399

[87] Cardinale D Colombo A Lamantia G Colombo N Civelli M De Giacomi G Anthracycline-induced cardiomyopathy: clinical relevance and response to pharmacologic therapy. J Am Coll Cardiol. (2010) 55(3):213–20. 10.1016/j.jacc.2009.03.095 20117401

[88] Dhesi S Chu MP Blevins G Paterson I Larratt L Oudit GY Cyclophosphamide-Induced cardiomyopathy: a case report, review, and recommendations for management. J Investig Med High Impact Case Rep. (2013) 1(1):2324709613480346. 10.1177/2324709613480346 26425570 PMC4528786

[89] Madondo MT Quinn M Plebanski M. Low dose cyclophosphamide: mechanisms of T cell modulation. Cancer Treat Rev. (2016) 42:3–9. 10.1016/j.ctrv.2015.11.005 26620820

[90] Gottdiener JS Appelbaum FR Ferrans VJ Deisseroth A Ziegler J. Cardiotoxicity associated with high-dose cyclophosphamide therapy. Arch Intern Med. (1981) 141(6):758–63. 10.1001/archinte.1981.00340060066015 7235784

[91] Wadia S. Acute cyclophosphamide hemorrhagic myopericarditis: dilemma case report, literature review and proposed diagnostic criteria. J Clin Diagn Res. (2015) 9(11):OE01–OE3. 10.7860/JCDR/2015/15054.6758 26674419 PMC4668459

[92] Ejaz K Raza MA Maroof S Haider MW. Cyclophosphamide-induced atrial fibrillation with rapid ventricular rate. Cureus. (2018) 10(5):e2633. 10.7759/cureus.2633 30034955 PMC6047839

[93] Goldberg MA Antin JH Guinan EC Rappeport JM. Cyclophosphamide cardiotoxicity: an analysis of dosing as a risk factor. Blood. (1986) 68(5):1114–8. 10.1182/blood.V68.5.1114.1114 3533179

[94] Katayama M Imai Y Hashimoto H Kurata M Nagai K Tamita K Fulminant fatal cardiotoxicity following cyclophosphamide therapy. J Cardiol. (2009) 54(2):330–4. 10.1016/j.jjcc.2009.01.006 19782276

[95] Taniguchi I. Clinical significance of cyclophosphamide-induced cardiotoxicity. Intern Med. (2005) 44(2):89–90. 10.2169/internalmedicine.44.89 15750266

[96] Braverman AC Antin JH Plappert MT Cook EF Lee RT. Cyclophosphamide cardiotoxicity in bone marrow transplantation: a prospective evaluation of new dosing regimens. J Clin Oncol. (1991) 9(7):1215–23. 10.1200/JCO.1991.9.7.1215 2045862

[97] Atalay F Gulmez O Ozsancak Ugurlu A. Cardiotoxicity following cyclophosphamidetherapy: a case report. J Med Case Rep. (2014) 8:252. 10.1186/1752-1947-8-252 25023062 PMC4106213

[98] Kyo M Ohshimo S Kida Y Shimatani T Torikoshi Y Suzuki K Pediatric cardiorespiratory failure successfully managed with venoarterial-venous extracorporeal membrane oxygenation: a case report. BMC Pulm Med. (2016) 16(1):119. 10.1186/s12890-016-0280-7 27519601 PMC4983066

[99] Iqubal A Iqubal MK Sharma S Ansari MA Najmi AK Ali SM Molecular mechanism involved in cyclophosphamide-induced cardiotoxicity: old drug with a new vision. Life Sci. (2019) 218:112–31. 10.1016/j.lfs.2018.12.018 30552952

[100] Michel G Socié G Gebhard F Bernaudin F Thuret I Vannier JP Late effects of allogeneic bone marrow transplantation for children with acute myeloblastic leukemia in first complete remission: the impact of conditioning regimen without total-body irradiation--a report from the Société Française de Greffe de Moelle. J Clin Oncol. (1997) 15(6):2238–46. 10.1200/JCO.1997.15.6.2238 9196136

[101] Petri M Brodsky R. High-dose cyclophosphamide and stem cell transplantation for refractory systemic lupus erythematosus. JAMA. (2006) 295(5):559–60. 10.1001/jama.295.5.559 16449623

[102] Traynor AE Schroeder J Rosa RM Cheng D Stefka J Mujais S Treatment of severe systemic lupus erythematosus with high-dose chemotherapy and haemopoietic stem-cell transplantation: a phase I study. Lancet. (2000) 356(9231):701–7. 10.1016/S0140-6736(00)02627-1 11085688

[103] Moyo VM Smith D Brodsky I Crilley P Jones RJ Brodsky RA. High-dose cyclophosphamide for refractory autoimmune hemolytic anemia. Blood. (2002) 100(2):704–6. 10.1182/blood-2002-01-0087 12091370

[104] Krishnan C Kaplin AI Brodsky RA Drachman DB Jones RJ Pham DL Reduction of disease activity and disability with high-dose cyclophosphamide in patients with aggressive multiple sclerosis. Arch Neurol. (2008) 65(8):1044–51. 10.1001/archneurol.65.8.noc80042 18541787 PMC2574697

[105] Verburg R Toes RE Fibbe WE Breedveld FC van Laar JM. High dose chemotherapy and autologous hematopoietic stem cell transplantation for rheumatoid arthritis: a review. Hum Immunol. (2002) 63(8):627–37. 10.1016/S0198-8859(02)00414-7 12121670

[106] Petri M Jones RJ Brodsky RA. High-dose cyclophosphamide without stem cell transplantation in systemic lupus erythematosus. Arthritis Rheum. (2003) 48(1):166–73. 10.1002/art.10752 12528116

[107] Kurauchi K Nishikawa T Miyahara E Okamoto Y Kawano Y. Role of metabolites of cyclophosphamide in cardiotoxicity. BMC Res Notes. (2017) 10(1):406. 10.1186/s13104-017-2726-2 28807058 PMC5557551

[108] Omole JG Ayoka OA Alabi QK Adefisayo MA Asafa MA Olubunmi BO Protective effect of kolaviron on cyclophosphamide-induced cardiac toxicity in rats. J Evid Based Integr Med. (2018) 23:2156587218757649. 10.1177/2156587218757649 29468886 PMC5871040

[109] Henning RJ Johnson GT Coyle JP Harbison RD. Acrolein can cause cardiovascular disease: a review. Cardiovasc Toxicol. (2017) 17(3):227–36. 10.1007/s12012-016-9396-5 28084565

[110] Ahlmann M Hempel G. The effect of cyclophosphamide on the immune system: implications for clinical cancer therapy. Cancer Chemother Pharmacol. (2016) 78(4):661–71. 10.1007/s00280-016-3152-1 27646791

[111] Ayza MA Zewdie KA Tesfaye BA Wondafrash DZ Berhe AH. The role of antioxidants in ameliorating cyclophosphamide-induced cardiotoxicity. Oxid Med Cell Longev. (2020) 2020:4965171. 10.1155/2020/4965171 32454939 PMC7238386

[112] Wang HT Chen TY Weng CW Yang CH Tang MS. Acrolein preferentially damages nucleolus eliciting ribosomal stress and apoptosis in human cancer cells. Oncotarget. (2016) 7(49):80450–64. 10.18632/oncotarget.12608 27741518 PMC5348333

[113] Aladaileh SH Abukhalil MH Saghir SAM Hanieh H Alfwuaires MA Almaiman AA Galangin activates Nrf2 signaling and attenuates oxidative damage, inflammation, and apoptosis in a rat model of cyclophosphamide-induced hepatotoxicity. Biomolecules. (2019) 9(8):346. 10.3390/biom9080346 31387329 PMC6723184

[114] El-Sheikh AA Morsy MA Okasha AM. Inhibition of NF-*κ*B/TNF-α pathway may be involved in the protective effect of resveratrol against cyclophosphamide-induced multi-organ toxicity. Immunopharmacol Immunotoxicol. (2017) 39(4):180–7. 10.1080/08923973.2017.1318913 28463035

[115] Nafees S Rashid S Ali N Hasan SK Sultana S. Rutin ameliorates cyclophosphamide induced oxidative stress and inflammation in Wistar rats: role of NF*κ*B/MAPK pathway. Chem Biol Interact. (2015) 231:98–107. 10.1016/j.cbi.2015.02.021 25753322

[116] Sayed-Ahmed MM Aldelemy ML Al-Shabanah OA Hafez MM Al-Hosaini KA Al-Harbi NO Inhibition of gene expression of carnitine palmitoyltransferase I and heart fatty acid binding protein in cyclophosphamide and ifosfamide-induced acute cardiotoxic rat models. Cardiovasc Toxicol. (2014) 14(3):232–42. 10.1007/s12012-014-9247-1 24469765

[117] Sayed-Ahmed MM Rishk AM Soloma S Abdel-aleem S. Protection by L-carnitine against the inhibition of gene expression of heart fatty acid binding protein by chronic administration of doxorubicin. Jour of the Egypt Nat. (2000) 12(4):275–81.

[118] Zhu M Liu Y Song Y Zhang S Hang C Wu F The role of METTL3-mediated N6-methyladenosine (m6A) of JPH2 mRNA in cyclophosphamide-induced cardiotoxicity. Front Cardiovasc Med. (2021) 8:763469. 10.3389/fcvm.2021.763469 34820430 PMC8606687

[119] Caruso G Privitera A Antunes BM Lazzarino G Lunte SM Aldini G The therapeutic potential of carnosine as an antidote against drug-induced cardiotoxicity and neurotoxicity: focus on Nrf2 pathway. Molecules. (2022) 27(14):4452. 10.3390/molecules27144452 35889325 PMC9324774

[120] Chakraborty M Bhattacharjee A Kamath JV. Cardioprotective effect of curcumin and piperine combination against cyclophosphamide-induced cardiotoxicity. Indian J Pharmacol. (2017) 49(1):65–70. 10.4103/0253-7613.201015 28458425 PMC5351241

[121] Nishikawa T Miyahara E Kurauchi K Watanabe E Ikawa K Asaba K Mechanisms of fatal cardiotoxicity following high-dose cyclophosphamide therapy and a method for its prevention. PLoS One. (2015) 10(6):e0131394. 10.1371/journal.pone.0131394 26114497 PMC4482695

[122] Refaie MMM Shehata S El-Hussieny M Abdelraheem WM Bayoumi AMA. Role of ATP-sensitive potassium channel (K_ATP_) and eNOS in mediating the protective effect of nicorandil in cyclophosphamide-induced cardiotoxicity. Cardiovasc Toxicol. (2020) 20(1):71–81. 10.1007/s12012-019-09535-8 31230218

[123] Stern S Liang D Li L Kurian R Lynch C Sakamuru S Targeting CAR and Nrf2 improves cyclophosphamide bioactivation while reducing doxorubicin-induced cardiotoxicity in triple-negative breast cancer treatment. JCI Insight. (2022) 7(12):e153868. 10.1172/jci.insight.153868 35579950 PMC9309041

[124] Siddik ZH. Cisplatin: mode of cytotoxic action and molecular basis of resistance. Oncogene. (2003) 22(47):7265–79. 10.1038/sj.onc.1206933 14576837

[125] Haugnes HS Wethal T Aass N Dahl O Klepp O Langberg CW Cardiovascular risk factors and morbidity in long-term survivors of testicular cancer: a 20-year follow-up study. J Clin Oncol. (2010) 28(30):4649–57. 10.1200/JCO.2010.29.9362 20855830

[126] Nuver J Smit AJ Sleijfer DT van Gessel AI van Roon AM van der Meer J Microalbuminuria, decreased fibrinolysis, and inflammation as early signs of atherosclerosis in long-term survivors of disseminated testicular cancer. Eur J Cancer. (2004) 40(5):701–6. 10.1016/j.ejca.2003.12.012 15010071

[127] Nuver J Smit AJ van der Meer J van den Berg MP van der Graaf WT Meinardi MT Acute chemotherapy-induced cardiovascular changes in patients with testicular cancer. J Clin Oncol. (2005) 23(36):9130–7. 10.1200/JCO.2005.01.4092 16301596

[128] Vaughn DJ Palmer SC Carver JR Jacobs LA Mohler ER. Cardiovascular risk in long-term survivors of testicular cancer. Cancer. (2008) 112(9):1949–53. 10.1002/cncr.23389 18338810

[129] Ma H Jones KR Guo R Xu P Shen Y Ren J. Cisplatin compromises myocardial contractile function and mitochondrial ultrastructure: role of endoplasmic reticulum stress. Clin Exp Pharmacol Physiol. (2010) 37(4):460–5. 10.1111/j.1440-1681.2009.05323.x 19878217

[130] Chalazan B Samra H Patel K Ash D Mehta A Konda S Cisplatin therapy as a risk factor for incident atrial fibrillation. Eur Heart J. (2019) 40(Suppl. 1):2447. 10.1093/eurheartj/ehz745.0121

[131] Sagcan F Citak EC Karpuz D Alakaya M. A rare entity: recurrent cisplatin-induced bradycardia. J Cancer Res Ther. (2020) 16(3):699–700. 10.4103/jcrt.JCRT_26_18 32719297

[132] Bang HJ Lee HY Kim HJ Yoon N Chung IJ Bae WK. Cisplatin-induced atrioventricular block requiring a pacemaker: two case reports and a literature review. Electrolyte Blood Press. (2020) 18(2):49–52. 10.5049/EBP.2020.18.2.49 33408748 PMC7781763

[133] Altena R Hummel YM Nuver J Smit AJ Lefrandt JD de Boer RA Longitudinal changes in cardiac function after cisplatin-based chemotherapy for testicular cancer. Ann Oncol. (2011) 22(10):2286–93. 10.1093/annonc/mdr408 21878427

[134] Wachters FM Van Der Graaf WT Groen HJ. Cardiotoxicity in advanced non-small cell lung cancer patients treated with platinum and non-platinum based combinations as first-line treatment. Anticancer Res. (2004) 24(3b):2079–83. 15274404

[135] Hu Y Sun B Zhao B Mei D Gu Q Tian Z. Cisplatin-induced cardiotoxicity with midrange ejection fraction: a case report and review of the literature. Medicine (Baltimore). (2018) 97(52):e13807. 10.1097/MD.0000000000013807 30593170 PMC6314747

[136] Ozben B Kurt R Oflaz H Sezer M Basaran M Goren T Acute anterior myocardial infarction after chemotherapy for testicular seminoma in a young patient. Clin Appl Thromb Hemost. (2007) 13(4):439–42. 10.1177/1076029607303334 17911198

[137] Ng KH Dearden C Gruber P. Rituximab-induced Takotsubo syndrome: more cardiotoxic than it appears? BMJ Case Rep. (2015) 2015:bcr2014208203. 10.1136/bcr-2014-208203 25733089 PMC4369036

[138] Shipa M Embleton-Thirsk A Parvaz M Santos LR Muller P Chowdhury K Effectiveness of belimumab after rituximab in systemic lupus erythematosus: a randomized controlled trial. Ann Intern Med. (2021) 174(12):1647–57. 10.7326/M21-2078 34698499

[139] Keating GM. Rituximab: a review of its use in chronic lymphocytic leukaemia, low-grade or follicular lymphoma and diffuse large B-cell lymphoma. Drugs. (2010) 70(11):1445–76. 10.2165/11201110-000000000-00000 20614951

[140] Foran JM Rohatiner AZ Cunningham D Popescu RA Solal-Celigny P Ghielmini M European Phase II study of rituximab (chimeric anti-CD20 monoclonal antibody) for patients with newly diagnosed mantle-cell lymphoma and previously treated mantle-cell lymphoma, immunocytoma, and small B-cell lymphocytic lymphoma. J Clin Oncol. (2000) 18(2):317–24. 10.1200/JCO.2000.18.2.317 10637245

[141] Arunprasath P Gobu P Dubashi B Satheesh S Balachander J. Rituximab induced myocardial infarction: a fatal drug reaction. J Cancer Res Ther. (2011) 7(3):346–8. 10.4103/0973-1482.87003 22044821

[142] Rabinovitz A Lombardo M Schinke C. Rituximab and cardiotoxicity. J Am Coll Cardiol. (2013):10(Suppl.):E582. 10.1016/S0735-1097(13)60582-3

[143] Coiffier B Lepage E Briere J Herbrecht R Tilly H Bouabdallah R CHOP Chemotherapy plus rituximab compared with CHOP alone in elderly patients with diffuse large-B-cell lymphoma. N Engl J Med. (2002) 346(4):235–42. 10.1056/NEJMoa011795 11807147

[144] Cheungpasitporn W Kopecky SL Specks U Bharucha K Fervenza FC. Non-ischemic cardiomyopathy after rituximab treatment for membranous nephropathy. J Renal Inj Prev. (2017) 6(1):18–25. 10.15171/jrip.2017.04 28487867 PMC5414514

[145] Madsen ML Due H Ejskjær N Jensen P Madsen J Dybkær K. Aspects of vincristine-induced neuropathy in hematologic malignancies: a systematic review. Cancer Chemother Pharmacol. (2019) 84(3):471–85. 10.1007/s00280-019-03884-5 31214762 PMC6682573

[146] Chatterjee K Zhang J Tao R Honbo N Karliner JS. Vincristine attenuates doxorubicin cardiotoxicity. Biochem Biophys Res Commun. (2008) 373(4):555–60. 10.1016/j.bbrc.2008.06.067 18590705 PMC2846088

[147] Merkx R Feijen ELAM Leerink JM de Baat EC Bellersen L van Dalen EC Cardiac function in childhood cancer survivors treated with vincristine: echocardiographic results from the DCCSS LATER 2 CARD study. Int J Cardiol. (2022) 369:69–76. 10.1016/j.ijcard.2022.07.049 35926643

[148] Gros R Hugon V Thouret JM Peigne V. Coronary spasm after an injection of vincristine. Chemotherapy. (2017) 62(3):169–71. 10.1159/000455224 28142134

[149] Goli AK Osman MN Koduri M Byrd RP Roy TM. A case report of vinorelbine monotherapy-related acute bronchospasm and non-ST elevation acute coronary syndrome. Tenn Med. (2011) 104(1):47–8. 21314064

[150] Dietrich J. Neurotoxicity of cancer therapies. Continuum (Minneap Minn). (2020) 26(6):1646–72. 10.1212/CON.0000000000000943 33273176

[151] Fyfe AJ McKay P. Toxicities associated with bleomycin. J R Coll Physicians Edinb. (2010) 40(3):213–5. 10.4997/JRCPE.2010.306 21127762

[152] Yamamoto T. Bleomycin and the skin. Br J Dermatol. (2006) 155(5):869–75. 10.1111/j.1365-2133.2006.07474.x 17034512

[153] Ishii H Takada K. Bleomycin induces E-selectin expression in cultured umbilical vein endothelial cells by increasing its mRNA levels through activation of NF-kappaB/Rel. Toxicol Appl Pharmacol. (2002) 184(2):88–97. 10.1006/taap.2002.9499 12408953

[154] Bokemeyer C Berger CC Kuczyk MA Schmoll HJ. Evaluation of long-term toxicity after chemotherapy for testicular cancer. J Clin Oncol. (1996) 14(11):2923–32. 10.1200/JCO.1996.14.11.2923 8918489

[155] White DA Schwartzberg LS Kris MG Bosl GJ. Acute chest pain syndrome during bleomycin infusions. Cancer. (1987) 59(9):1582–5. 10.1002/1097-0142(19870501)59:9<1582::AID-CNCR2820590909>3.0.CO;2-# 2435402

[156] Didagelos M Boutis A Diamantopoulos N Sotiriadou M Fotiou C. Bleomycin cardiotoxicity during chemotherapy for an ovarian germ cell tumor. Hippokratia. (2013) 17(2):187–8. 24376332 PMC3743631

[157] Gozhenko A Bestanchuk O Kaschenko O Narbutova T. Cumulative cardiotoxic effect of bleomycin in experiment. J Educ Health Sport. (2021) 11:301–8. 10.12775/JEHS.2021.11.06.033

[158] Sanada M Hidaka M Takagi Y Takano TY Nakatsu Y Tsuzuki T Modes of actions of two types of anti-neoplastic drugs, dacarbazine and ACNU, to induce apoptosis. Carcinogenesis. (2007) 28(12):2657–63. 10.1093/carcin/bgm188 17881774

[159] Etebari M Jafarian-Dehkordi A Lame V. Evaluation of protective effect of amifostine on dacarbazine induced genotoxicity. Res Pharm Sci. (2015) 10(1):68–74. 26430459 PMC4578214

[160] Montecucco A Zanetta F Biamonti G. Molecular mechanisms of etoposide. Excli J. (2015) 14:95–108. 10.17179/excli2015-561 26600742 PMC4652635

[161] Meresse P Dechaux E Monneret C Bertounesque E. Etoposide: discovery and medicinal chemistry. Curr Med Chem. (2004) 11(18):2443–66. 10.2174/0929867043364531 15379707

[162] Escoto H Ringewald J Kalpatthi R. Etoposide-related cardiotoxicity in a child with haemophagocytic lymphohistiocytosis. Cardiol Young. (2010) 20(1):105–7. 10.1017/S1047951109991272 20181298

[163] Akam-Venkata J Franco VI Lipshultz SE. Late cardiotoxicity: issues for childhood cancer survivors. Curr Treat Options Cardiovasc Med. (2016) 18(7):47. 10.1007/s11936-016-0466-6 27230282

[164] Gill D Mann K Kaur S Ruiz VG. Rare cause of cardiotoxicity. Arch Med. (2017) 9(1):1–2. 10.21767/1989-5216.1000193

[165] Kridis WB Khanfir A Triki F Frikha M. An exceptional case of atrial fibrillation arrhythmia induced by etoposide. Curr Drug Saf. (2013) 8(4):287–9. 10.2174/15748863113080990047 23962185

[166] Ozkan HA Bal C Gulbas Z. Assessment and comparison of acute cardiac toxicity during high-dose cyclophosphamide and high-dose etoposide stem cell mobilization regimens with N-terminal pro-B-type natriuretic peptide. Transfus Apher Sci. (2014) 50(1):46–52. 10.1016/j.transci.2013.12.001 24382557

[167] Shabana S Aden S Abdulrahman N Riaz S. The efficacy of etoposide on H9c2 cardiomyoblasts against doxorubicin induced cardiotoxicity. Anat Physiol. (2015) 5(4):1–5. 10.4172/2161-0940.1000186

[168] Olayinka ET Ore A Adeyemo OA Ola OS Olotu OO Echebiri RC. Quercetin, a flavonoid antioxidant, ameliorated procarbazine-induced oxidative damage to murine tissues. Antioxidants (Basel). (2015) 4(2):304–21. 10.3390/antiox4020304 26783707 PMC4665474

[169] Cruz-Topete D Myers PH Foley JF Willis MS Cidlowski JA. Corticosteroids are essential for maintaining cardiovascular function in male mice. Endocrinology. (2016) 157(7):2759–71. 10.1210/en.2015-1604 27219275 PMC4929548

[170] Krishnamoorthy A Mentz RJ Hyland KA McMillan EB Patel CB Milano CA A crisis of the heart: an acute reversible cardiomyopathy bridged to recovery in a patient with Addison’s disease. ASAIO J. (2013) 59(6):668–70. 10.1097/MAT.0000000000000001 24172274

[171] Vasheghani-Farahani A Sahraian MA Darabi L Aghsaie A Minagar A. Incidence of various cardiac arrhythmias and conduction disturbances due to high dose intravenous methylprednisolone in patients with multiple sclerosis. J Neurol Sci. (2011) 309(1–2):75–8. 10.1016/j.jns.2011.07.018 21831398

[172] Taylor MR Gaco D. Symptomatic sinus bradycardia after a treatment course of high-dose oral prednisone. J Emerg Med. (2013) 45(3):e55–8. 10.1016/j.jemermed.2013.04.020 23827163

[173] Nagakura A Morikawa Y Sakakibara H Miura M. Bradycardia associated with prednisolone in children with severe kawasaki disease. J Pediatr. (2017) 185:106–11.e1. 10.1016/j.jpeds.2017.02.074 28343657

[174] Al Shibli A Al Attrach I Hamdan MA. Bradycardia following oral corticosteroid use: case report and literature review. Arab J Nephrol Transplant. (2012) 5(1):47–9. 22283866

[175] Khandelwal K Madathala RR Chennaiahgari N Yousuffuddin M. Steroid-induced sinus bradycardia. Cureus. (2021) 13(5):e15065. 10.7759/cureus.15065 34141509 PMC8205859

[176] Üsküdar Cansu D Bodakçi E Korkmaz C. Dose-dependent bradycardia as a rare side effect of corticosteroids: a case report and review of the literature. Rheumatol Int. (2018) 38(12):2337–43. 10.1007/s00296-018-4167-1 30276424

[177] John PR Khaladj-Ghom A Still KL. Bradycardia associated with steroid use for laryngeal edema in an adult: a case report and literature review. Case Rep Cardiol. (2016) 2016:9785467. 10.1155/2016/9785467 27999689 PMC5143689

[178] Schellong G Riepenhausen M Bruch C Kotthoff S Vogt J Bölling T Late valvular and other cardiac diseases after different doses of mediastinal radiotherapy for Hodgkin disease in children and adolescents: report from the longitudinal GPOH follow-up project of the German-Austrian DAL-HD studies. Pediatr Blood Cancer. (2010) 55(6):1145–52. 10.1002/pbc.22664 20734400

[179] Maraldo MV Brodin NP Vogelius IR Aznar MC Munck Af Rosenschöld P Petersen PM Risk of developing cardiovascular disease after involved node radiotherapy versus mantle field for Hodgkin lymphoma. Int J Radiat Oncol Biol Phys. (2012) 83(4):1232–7. 10.1016/j.ijrobp.2011.09.020 22270170

[180] Horimoto M Igarashi K Takenaka T Batra S. Pulmonary infundibular stenosis, coronary artery disease, and aortic regurgitation caused by mediastinal radiation. Am Heart J. (1993) 126(4):1002–5. 10.1016/0002-8703(93)90723-M 8213419

[181] Lund MB Ihlen H Voss BM Abrahamsen AF Nome O Kongerud J Increased risk of heart valve regurgitation after mediastinal radiation for Hodgkin’s disease: an echocardiographic study. Heart. (1996) 75(6):591–5. 10.1136/hrt.75.6.591 8697163 PMC484383

[182] Bijl JM Roos MM van Leeuwen-Segarceanu EM Vos JM Bos WW Biesma DH Assessment of valvular disorders in survivors of hodgkin’s lymphoma treated by mediastinal radiotherapy ± chemotherapy. Am J Cardiol. (2016) 117(4):691–6. 10.1016/j.amjcard.2015.11.027 26772441

[183] Heidenreich PA Hancock SL Lee BK Mariscal CS Schnittger I. Asymptomatic cardiac disease following mediastinal irradiation. J Am Coll Cardiol. (2003) 42(4):743–9. 10.1016/S0735-1097(03)00759-9 12932613

[184] Stewart FA Heeneman S Te Poele J Kruse J Russell NS Gijbels M Ionizing radiation accelerates the development of atherosclerotic lesions in ApoE-/- mice and predisposes to an inflammatory plaque phenotype prone to hemorrhage. Am J Pathol. (2006) 168(2):649–58. 10.2353/ajpath.2006.050409 16436678 PMC1606487

[185] McEniery PT Dorosti K Schiavone WA Pedrick TJ Sheldon WC. Clinical and angiographic features of coronary artery disease after chest irradiation. Am J Cardiol. (1987) 60(13):1020–4. 10.1016/0002-9149(87)90345-6 3673902

[186] Brosius FC Waller BF Roberts WC. Radiation heart disease. Analysis of 16 young (aged 15 to 33 years) necropsy patients who received over 3,500 rads to the heart. Am J Med. (1981) 70(3):519–30. 10.1016/0002-9343(81)90574-X 6782873

[187] van den Belt-Dusebout AW Nuver J de Wit R Gietema JA ten Bokkel Huinink WW Rodrigus PT Long-term risk of cardiovascular disease in 5-year survivors of testicular cancer. J Clin Oncol. (2006) 24(3):467–75. 10.1200/JCO.2005.02.7193 16421423

[188] Řiháčková E Elbl L Řiháček M Holická M Kala P. Anti-cancer therapy-induced metabolic syndrome. Vnitr Lek. (2021) 67(6):334–8. 10.36290/vnl.2021.089 35459375

[189] van Nimwegen FA Schaapveld M Cutter DJ Janus CP Krol AD Hauptmann M Radiation dose-response relationship for risk of coronary heart disease in survivors of hodgkin lymphoma. J Clin Oncol. (2016) 34(3):235–43. 10.1200/JCO.2015.63.4444 26573075

[190] Chello M Mastroroberto P Romano R Zofrea S Bevacqua I Marchese AR. Changes in the proportion of types I and III collagen in the left ventricular wall of patients with post-irradiative pericarditis. Cardiovasc Surg. (1996) 4(2):222–6. 10.1016/0967-2109(96)82320-9 8861442

[191] Cohen SI Bharati S Glass J Lev M. Radiotherapy as a cause of complete atrioventricular block in hodgkin’s disease. An electrophysiological-pathological correlation. Arch Intern Med. (1981) 141(5):676–9. 10.1001/archinte.1981.00340050122029 7224752

[192] Modi S Baig W. Radiotherapy-induced Tako-tsubo cardiomyopathy. Clin Oncol (R Coll Radiol). (2009) 21(4):361–2. 10.1016/j.clon.2009.01.005 19230629

[193] Giyanani N Som S. When too many hits break the heart: a case of radiation induced takotsubo cardiomyopathy. Am J Med Sci. (2021) 362(2):215–9. 10.1016/j.amjms.2021.03.014 33819487

[194] Galper SL Yu JB Mauch PM Strasser JF Silver B Lacasce A Clinically significant cardiac disease in patients with Hodgkin lymphoma treated with mediastinal irradiation. Blood. (2011) 117(2):412–8. 10.1182/blood-2010-06-291328 20858859

[195] Chow EJ Chen Y Hudson MM Feijen EAM Kremer LC Border WL Prediction of ischemic heart disease and stroke in survivors of childhood cancer. J Clin Oncol. (2018) 36(1):44–52. 10.1200/JCO.2017.74.8673 29095680 PMC5756324

[196] Vordermark D Seufert I Schwab F Kölbl O Kung M Angermann C 3-D Reconstruction of anterior mantle-field techniques in Hodgkin’s disease survivors: doses to cardiac structures. Radiat Oncol. (2006) 1:10. 10.1186/1748-717X-1-10 16722610 PMC1464386

[197] Zamorano JL Gottfridsson C Asteggiano R Atar D Badimon L Bax JJ The cancer patient and cardiology. Eur J Heart Fail. (2020) 22(12):2290–309. 10.1002/ejhf.1985 32809231 PMC8278961

[198] Barlaz Us S Vezir O Yildirim M Bayrak G Yalin S Balli E Protective effect of N-acetyl cysteine against radiotherapy-induced cardiac damage. Int J Radiat Biol. (2020) 96(5):661–70. 10.1080/09553002.2020.1721605 31990607

[199] Wang L Qin W Huo YJ Li X Shi Q Rasko JEJ Advances in targeted therapy for malignant lymphoma. Signal Transduct Target Ther. (2020) 5(1):15. 10.1038/s41392-020-0113-2 32296035 PMC7058622

[200] Dolladille C Ederhy S Allouche S Dupas Q Gervais R Madelaine J Late cardiac adverse events in patients with cancer treated with immune checkpoint inhibitors. J Immunother Cancer. (2020) 8(1):1–9. 10.1136/jitc-2019-000261 PMC7057417 31988143

[201] McMullen JR Boey EJ Ooi JY Seymour JF Keating MJ Tam CS. Ibrutinib increases the risk of atrial fibrillation, potentially through inhibition of cardiac PI3K-Akt signaling. Blood. (2014) 124(25):3829–30. 10.1182/blood-2014-10-604272 25498454

[202] Arustamyan M Kibrik P Hatipoglu D Bungo B Mentias A Hill BT The safety of Bruton’s tyrosine kinase inhibitors in B-cell malignancies: a systematic review. Eur J Haematol. (2022) 109(6):696–710. 10.1111/ejh.13854 36030394

[203] Mato AR Shah NN Jurczak W Cheah CY Pagel JM Woyach JA Pirtobrutinib in relapsed or refractory B-cell malignancies (BRUIN): a phase 1/2 study. Lancet. (2021) 397(10277):892–901. 10.1016/S0140-6736(21)00224-5 33676628 PMC11758240

[204] Chen X Jiang A Zhang R Fu X Liu N Shi C Immune checkpoint inhibitor-associated cardiotoxicity in solid tumors: real-world incidence, risk factors, and prognostic analysis. Front Cardiovasc Med. (2022) 9:882167. 10.3389/fcvm.2022.882167 35669482 PMC9163804

[205] Lin N Song Y Zhu J. Immune checkpoint inhibitors in malignant lymphoma: advances and perspectives. Chin J Cancer Res. (2020) 32(3):303–18. 10.21147/j.issn.1000-9604.2020.03.03 32694896 PMC7369179

[206] Johnson DB Balko JM Compton ML Chalkias S Gorham J Xu Y Fulminant myocarditis with combination immune checkpoint blockade. N Engl J Med. (2016) 375(18):1749–55. 10.1056/NEJMoa1609214 27806233 PMC5247797

[207] Nykl R Fischer O Vykoupil K Taborsky M. A unique reason for coronary spasm causing temporary ST elevation myocardial infarction (inferior STEMI)—systemic inflammatory response syndrome after use of pembrolizumab. Arch Med Sci Atheroscler Dis. (2017) 2:e100–2. 10.5114/amsad.2017.72531 29379889 PMC5777475

[208] Ganatra S Neilan TG. Immune checkpoint inhibitor-associated myocarditis. Oncologist. (2018) 23(8):879–86. 10.1634/theoncologist.2018-0130 29802219 PMC6156176

[209] Palaskas N Lopez-Mattei J Durand JB Iliescu C Deswal A. Immune checkpoint inhibitor myocarditis: pathophysiological characteristics, diagnosis, and treatment. J Am Heart Assoc. (2020) 9(2):e013757. 10.1161/JAHA.119.013757 31960755 PMC7033840

[210] Camilli M Maggio L Tinti L Lamendola P Lanza GA Crea F Chimeric antigen receptor-T cell therapy-related cardiotoxicity in adults and children cancer patients: a clinical appraisal. Front Cardiovasc Med. (2023) 10:1090103. 10.3389/fcvm.2023.1090103 36895831 PMC9988907

[211] Rao A Stewart A Eljalby M Ramakrishnan P Anderson LD Awan FT Cardiovascular disease and chimeric antigen receptor cellular therapy. Front Cardiovasc Med. (2022) 9:932347. 10.3389/fcvm.2022.932347 36211558 PMC9538377

[212] Lee DH Chandrasekhar S Jain M Chavez J Shah B Lazaryan A Abstract 9828: active surveillance of cardiotoxicity with cardiac biomarkers during chimeric antigen receptor T-cell therapy. Circulation. (2021) 144(Suppl_1):A9828-A.

[213] Totzeck M Michel L Lin Y Herrmann J Rassaf T. Cardiotoxicity from chimeric antigen receptor-T cell therapy for advanced malignancies. Eur Heart J. (2022) 43(20):1928–40. 10.1093/eurheartj/ehac106 35257157 PMC9123242

[214] ClinicalTrials.gov. Bethesda (MD): National Library of Medicine (US). 2000 Feb 29 -. Identifier NCT02943590, STOP-CA (Statins TO Prevent the Cardiotoxicity From Anthracyclines) (2022). Available at: https://clinicaltrials.gov/ct2/show/study/NCT02943590?term=NCT02943590&draw=2&rank=1 (Cited 2023 May 8).

[215] Neilan TG. The STOP-CA trial. Presented at: ACC/WCC 2023. March 4, 2023.:New Orleans, LA.

[216] ClinicalTrials.gov. Bethesda (MD): National Library of Medicine (US). 2000 Feb 29 -. Identifier NCT02818517, Evaluation and Management of Cardio Toxicity in Oncologic Patients. (2022). Available at: https://clinicaltrials.gov/ct2/show/NCT02818517?term=NCT02818517&draw=2&rank=1 (Cited 2023 May 8).

[217] Stein-Merlob AF Rothberg MV Holman P Yang EH. Immunotherapy-Associated cardiotoxicity of immune checkpoint inhibitors and chimeric antigen receptor T cell therapy: diagnostic and management challenges and strategies. Curr Cardiol Rep. (2021) 23(3):11. 10.1007/s11886-021-01440-3 33483873 PMC7821837

[218] Yu S Yi M Qin S Wu K. Next generation chimeric antigen receptor T cells: safety strategies to overcome toxicity. Mol Cancer. (2019) 18(1):125. 10.1186/s12943-019-1057-4 31429760 PMC6701025

[219] Alvi RM Frigault MJ Fradley MG Jain MD Mahmood SS Awadalla M Cardiovascular events among adults treated with chimeric antigen receptor T-cells (CAR-T). J Am Coll Cardiol. (2019) 74(25):3099–108. 10.1016/j.jacc.2019.10.038 31856966 PMC6938409

[220] Yarmarkovich M Marshall QF Warrington JM Premaratne R Farrel A Groff D Cross-HLA targeting of intracellular oncoproteins with peptide-centric CARs. Nature. (2021) 599(7885):477–84. 10.1038/s41586-021-04061-6 34732890 PMC8599005

[221] Flugel CL Majzner RG Krenciute G Dotti G Riddell SR Wagner DL Overcoming on-target, off-tumour toxicity of CAR T cell therapy for solid tumours. Nat Rev Clin Oncol. (2023) 20(1):49–62. 10.1038/s41571-022-00704-3 36418477 PMC10278599

[222] Cameron BJ Gerry AB Dukes J Harper JV Kannan V Bianchi FC Identification of a titin-derived HLA-A1-presented peptide as a cross-reactive target for engineered MAGE A3-directed T cells. Sci Transl Med. (2013) 5(197):197ra03. 10.1126/scitranslmed.3006034 PMC6002776 23926201

[223] Abbott RC Hughes-Parry HE Jenkins MR. To go or not to go? Biological logic gating engineered T cells. J Immunother Cancer. (2022) 10(4):1–10. 10.1136/jitc-2021-004185 PMC8981284 35379738

[224] Keam SJ. Pirtobrutinib: first approval. Drugs. (2023) 83(6):547–53. 10.1007/s40265-023-01860-1 37004673

